# Improving oral health and related health behaviours (substance use, smoking, diet) in people with severe and multiple disadvantage: A systematic review of effectiveness and cost-effectiveness of interventions

**DOI:** 10.1371/journal.pone.0298885

**Published:** 2024-04-18

**Authors:** Laura J. McGowan, Deepti A. John, Ryan P. W. Kenny, Emma C. Joyes, Emma A. Adams, Hosein Shabaninejad, Catherine Richmond, Fiona R. Beyer, David Landes, Richard G. Watt, Falko F. Sniehotta, Martha Paisi, Claire Bambra, Dawn Craig, Eileen Kaner, Sheena E. Ramsay

**Affiliations:** 1 Population Health Sciences Institute, Newcastle University, Newcastle upon Tyne, United Kingdom; 2 Evidence Synthesis Group and Innovation Observatory, Population Health Sciences Institute, Newcastle University, Newcastle Upon Tyne, United Kingdom; 3 NHS England & Improvement, Newcastle Upon Tyne, United Kingdom; 4 Department of Epidemiology and Public Health, University College London, London, United Kingdom; 5 NIHR Policy Research Unit Behavioural Science, Newcastle University, Newcastle Upon Tyne, United Kingdom; 6 Department of Public Health, Social and Preventive Medicine, Centre for Preventive Medicine and Digital Health (CPD), Medical Faculty Mannheim, Heidelberg University, Mannheim, Germany; 7 Faculty of Medicine and Dentistry, Peninsula Dental School, University of Plymouth, Plymouth, United Kingdom; NYU Grossman School of Medicine: New York University School of Medicine, UNITED STATES

## Abstract

**Background:**

People experiencing homelessness co-occurring with substance use or offending (‘severe and multiple disadvantage’ SMD) often have high levels of poor oral health and related health behaviours (particularly, substance use, smoking, poor diet). This systematic review aimed to assess the effectiveness and cost-effectiveness of interventions in adults experiencing SMD to improve oral health and related health behaviours.

**Methods and findings:**

From inception to February 2023, five bibliographic databases (MEDLINE, EMBASE, PsycINFO, CINAHL, and Scopus) and grey literature were searched. Two researchers independently screened the search results. Randomized controlled trials (RCTs), comparative studies and economic evaluations were included that reported outcomes on oral health and the related health behaviours. Risk of bias was assessed and results narratively synthesized. Meta-analyses were performed where appropriate. This review was registered with PROSPERO (reg. no: CRD42020202416). Thirty-eight studies were included (published between 1991 and 2023) with 34 studies reporting about effectiveness. Most studies reported on substance use (n = 30). Interventions with a combination of housing support with substance use and mental health support such as contingent work therapy appeared to show some reduction in substance use in SMD groups. However, meta-analyses showed no statistically significant results. Most studies had short periods of follow-up and high attrition rates. Only one study reported on oral health; none reported on diet. Three RCTs reported on smoking, of which one comprising nicotine replacement with contingency management showed improved smoking abstinence at 4 weeks compared to control. Five studies with economic evaluations provided some evidence that interventions such as Housing First and enhanced support could be cost-effective in reducing substance use.

**Conclusion:**

This review found that services such as housing combined with other healthcare services could be effective in improving health behaviours, particularly substance use, among SMD groups. Gaps in evidence also remain on oral health improvement, smoking, and diet. High quality studies on effectiveness with adequate power and retention are needed to address these significant health challenges in SMD populations.

## Introduction

High income countries such as the UK have an increasing number of people experiencing homelessness [1]. This includes not only ‘rough sleeping on the streets or outside’ but all forms of homelessness, for example, sleeping on sofas of friends and family (sofa surfing) and temporary accommodation [2]. People experiencing homelessness also face overlapping challenges of substance use and frequent involvement with the criminal justice system–together these issues have been termed as ‘severe and multiple disadvantage’ (SMD) [2].

Populations experiencing homelessness and SMD in the UK and globally have some of the highest levels of physical and mental ill-health, and lowest life expectancy in the population [3]. Poor oral health is one of the most commonly reported health problems in SMD groups [3]. SMD groups have extremely high levels of tooth loss, decay, infections and pain [4]. Poor dental health in turn impacts on confidence, mental health, self-esteem and it could be a factor in perpetuating homelessness [5]. Importantly, poor oral health is integrally linked with drug and alcohol use, smoking, and poor diet, which are also disproportionately high in SMD groups [4, 6]. Tackling these health behaviours is important for improving oral health outcomes in SMD groups [7]. Previous research by the charity, Groundswell, has shown that approximately 90% of people experiencing SMD have encountered oral health issues often resorting to self-treatment and self- medication with drugs and alcohol. Additionally, this population was known to have heightened levels of sugar consumption and tobacco use further increasing their susceptibility to oral health issues [5].

Whilst a large body of evidence is available on the burden of oral ill-health and related health behaviours in SMD groups, there is relatively less evidence on interventions that are effective in improving these outcomes [5, 6, 8]. Interventions can be conceptualised at structural, community and individual levels to tackle the different determinants of health, including oral health and behaviours [9]. Previous reviews have summarised evidence on substance use interventions centred on homelessness and housing related components [10]. There remains a need to synthesise the evidence on interventions for the related behavioural risk factors of substance use, smoking, diet, along with oral health outcomes. Moreover, there is a recognition that interventions to improve health outcomes need to address the overlapping needs of SMD groups, beyond housing/homelessness [11]. The recent National Institute for Health and Care Excellence (NICE) guidelines on homelessness in England reported that by integrating health and social care access, engagement by SMD groups could be improved [12]. The aim of this paper was to review the effectiveness (and cost-effectiveness) of interventions in improving oral health and related health behaviours (smoking, substance use, and diet) in adults experiencing SMD.

## Methods

This systematic review is reported according to Preferred Reporting Items for Systematic Reviews and Meta-Analyses (PRISMA) guidelines (S1 Checklist). The protocol was pre-registered on PROSPERO (CRD42020202416) [13, 14]. The review was underpinned by our initial logic model (S1 File), which drew on early scoping of literature, input from people with experience of SMD, and the collective knowledge of the research team. The logic model helped define the scope of the review and guided conceptualisation throughout [15]. To enhance the relevance of review findings to the target population, the review was undertaken with input from those with experience of SMD (‘Experts by Experience’) and a range of stakeholders from policy and practice (including voluntary sector, local authority, health services, policy makers) [16–18]. An ‘Experts by Experience’ network from a local charity in Newcastle/ Gateshead provided input into the review. The network includes people with lived experience of SMD (including, homelessness, substance use or offending behaviour). At different stages of the review 2–4 people from this network provided input, including in the scope of the review, particularly on understanding interventions related to substance use and criminal justice systems, and in sense-making of emerging findings. ‘Experts by Experience’ and stakeholders from policy provided insights on outcomes of relevance to populations and policy makers, on early findings and interpretation of results.

### Searches and screening

Studies were included if they met the inclusion criteria detailed in Table 1. Primary outcomes included oral health (e.g., dental caries, periodontal disease, tooth loss, pain, and dental infections), health behaviours (drug and/or alcohol use, smoking, and diet), economic outcomes (cost-effectiveness, costs, resource use) (Table 1). A single search strategy was used for the effectiveness and cost-effectiveness aspects of the review. The search strategy was developed using a combination of keyword and subject headings in MEDLINE (S2 File) [14]. The following databases were searched from inception until February 2023: EMBASE (Ovid); CINAHL (Ebsco); APA PsycINFO (Ovid); and Scopus. Forward and backward citation searching was conducted on included studies. Grey literature searches were conducted using Google Incognito and relevant charity and organisation websites (e.g. Fulfilling Lives, Crisis).

**Table 1:** Inclusion criteria for the systematic review.

**Population and Setting**	Adults (≥18 years) with SMD comprising homelessness (rough sleeping and other forms of highly insecure/inadequate accommodation); repeat offending (persistent, low-level offending); or problematic substance use (use of drugs or alcohol in a harmful way) where this co-occurs with homelessness and/or repeat offending.
**Interventions**	Any interventions at structural, community and individual levels e.g. structural-level interventions related to housing, co-locating services for housing, rehabilitation, employment and healthcare; community-level interventions including oral health promotion in temporary housing; and individual-level interventions such as, rehabilitation services, motivational interviewing, peer-support.
**Comparators**	Studies that compared an eligible intervention against any comparator, such as, standard care. Studies with no comparator (i.e., single arm studies) were not eligible for inclusion.
**Outcomes**	Eligible primary outcomes were oral health (e.g., dental caries, periodontal disease, tooth loss etc.), health behaviours (diet, smoking, substance use, including drug and/or alcohol use/dependence), economic outcomes (cost-effectiveness, costs, resource use), and adverse effects. Secondary outcomes included mental wellbeing, health-related quality of life, self-esteem, employment, and income. Studies had to report at least one of the primary outcomes to be eligible for inclusion.
**Study Design**	All comparative study designs were included, e.g. individual and cluster randomised controlled trials (RCTs), quasi-experimental studies, prospective, and comparative non-RCTs (e.g., cohort studies and case control studies). Comparative economic evaluations reporting costs of interventions were also eligible, including cost-minimization analysis (CMA), cost-effectiveness analysis (CEA), cost–benefit analysis (CBA), cost–utility analysis (CUA), model-based economic evaluation, and interrupted time series (ITS). Pilot/feasibility or single-arm studies, systematic and non-systematic reviews, individual case reports, commentaries, editorials, letters, and opinion pieces were ineligible for either review. Any studies published as abstracts or conference presentations were eligible for inclusion, provided that any outcome data of interest were sufficiently reported.
**Limits**	No limit on date, country or language was applied. Translations for papers in other languages will be attempted, if not possible they will be included as an appendix for completeness.

References generated from the search were uploaded to Endnote and de-duplicated. Screening was managed in Covidence [19]. Title/abstract and full-text screening was conducted independently by two reviewers. Discrepancies were resolved through discussion and consulting a third reviewer to reach consensus.

### Data analysis

Data extraction and quality assessment was conducted by one reviewer and double checked by a second reviewer. Study information extracted is detailed in Table 2. Randomised controlled trials (RCTs) were assessed using the Cochrane risk-of-bias tool [20]. Non-randomised studies were appraised using the Critical Appraisal Skills Program (CASP) cohort study checklist [21]. Grading of Recommendations, Assessment, Development and Evaluation (GRADE) was applied for meta-analyses of RCTs (S3 File) [22]. Economic evaluations were assessed using the BMJ checklist (S4 File) [23]. Studies were not excluded on the basis of quality; rather quality assessment was used to aid interpretation of analysis. Any discrepancies in extraction or quality assessment were resolved through discussion and consultation with a third reviewer.

**Table 2:** Data extraction items.

**Identification**	Covidence ID; author name; year; title; publication type; country; funding source; conflicts of interest
**Methods**	Study aim; design; number of arms; unit of allocation (e.g. individuals/groups); primary outcome category (oral health, smoking, diet, substance use, mixed); imputation of missing data; unit of analysis (e.g. individuals, practice, community); type of comparison
**Participants**	Population group (homeless, substance users, repeat offenders, mixed); population group type (e.g. specific type of homelessness/substance use); key inclusion/exclusion criteria; total number of participants; baseline imbalances; age; sex; ethnicity; education; employment; housing status
**Intervention details**	Items based on the template for intervention description and replication [TIDieR]: WHY (rationale; use of theory; use of explicit behaviour change techniques); WHAT (materials used, procedures followed, use of co-production); WHO PROVIDED (who delivered intervention and description of background/expertise); HOW (mode of delivery, individual or group provision); WHERE (setting, incl. description); WHEN & HOW MUCH (number of sessions, their schedule, and their duration, intensity or dose); MODIFICATIONS (any changes to intervention over the course of the study); HOW WELL (assessment of intervention adherence or fidelity [planned and actual])
**Cost and resources**	Perspective; time horizon; cost values; currency used; resource requirements; years to which costs apply; methods of handling cost data; type of economic evaluation; model type; uncertainty around economic evaluation analysis
**Results**	Primary outcomes (related to review); secondary outcomes (related to review); timepoints; statistical analysis; dichotomous outcomes–number of participants/no. of events for intervention and control groups, other results reported (e.g. odds ratio, risk difference, p values, confidence intervals); continuous outcomes–number of participants, means, standard deviations for intervention and control groups, other results reported (e.g. standardised mean difference, F values, p values, confidence intervals); number of missing participants; number of participants moved to other group; adverse outcomes

### Data synthesis

Findings on effectiveness of interventions were narratively synthesised for primary outcomes. The synthesis explored outcome measures, intervention types and study quality. To synthesise results for substance use related outcomes, each specific outcome (drugs, alcohol, and combined substance use including drugs and alcohol) was categorised into three groups of measures: measures of addiction-related problems, frequency-related measures, and ‘other’. Addiction-related measures were indicative of severity of drug and alcohol-related problems and encompassed issues associated with drug/alcohol use, including employment, legal, and family/social issues, and level of craving (e.g., physical and psychological symptoms). Frequency-related measures encompassed how often drugs/alcohol were consumed (e.g., number of days of drug/alcohol/substance use or number of days of abstinence)–these measures are indicative of addition or dependence. ‘Other’ measures included money spent on drugs/alcohol, number of different drugs used, highest frequency of drugs used, peak alcohol quantity, and treatment progression.

Meta-analysis was performed in R [24], on studies reporting frequency of drug use (or abstinence) outcomes. These outcomes were the most homogenous and conceptually similar measures and were also deemed by stakeholders (policy makers, ‘Experts by Experience’) to be of particular relevance. It was not feasible to undertake meta-analyses of other measures reported due to heterogeneity. Data were converted to standardised mean difference (SMD, Cohen’s *D*) scores, using the esc package [25, 26]. For dichotomous data, odds ratios were calculated then converted to SMDs [25]. Data from studies reporting frequency of abstinence were reversed to ensure data for each study represented frequency of drug consumption rather than abstinence. Where studies assessed more than one intervention, data were extracted for any comparisons that were relevant to the review, avoiding double-counting by dividing the number of participants in the control arm evenly between comparisons.

Authors were contacted requesting any relevant missing data for studies conducted in the past 15 years. For missing standard deviations, where data could not be obtained from study authors, we imputed estimates by pooling standard deviations from other similar studies with the same type of outcome/measure. Heterogeneity was assessed using I^2^ and tau (τ) statistics using the restricted maximum likelihood (REML) method. Additionally, the Hartung-Knapp correction was used to correct for the small number of studies included in each analysis [27]. Furthermore, we present prediction intervals, which represent the expected range of true effects in similar studies [28]. Where multiple time points were reported for short-, medium- or long-term outcomes (e.g. 3 and 6 months for short-term outcomes), we combined the time points using the formula recommended by Borenstein et al. [29]. For the calculation of variance, we assumed a high correlation between the time points of 0.8. Narrative synthesis of findings from economic evaluations reported in included studies was undertaken.

## Results

**Fig 1:**
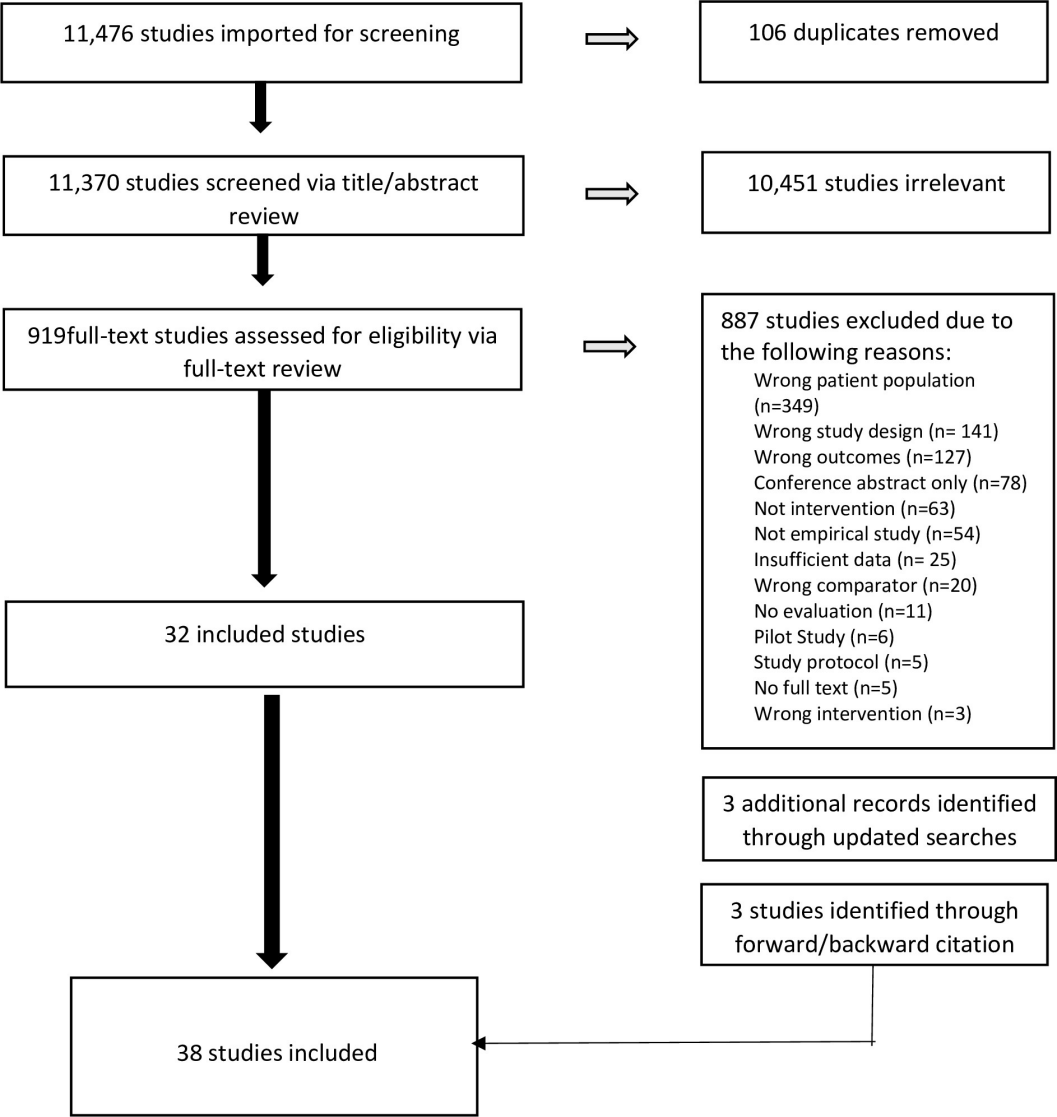
PRISMA flow diagram of studies included at each stage of the screening process.

Database searches identified 11,476 unique studies; none from grey literature (Fig 1). Following abstract and full-text screening, 34 studies were eligible for inclusion in the effectiveness review, and 5 in the cost-effectiveness analysis with one study that was included in both since it had relevant findings.

### Characteristics of included studies

Key characteristics of the included unique studies are summarised in Table 3. Studies were published between 1991 and 2023. Most studies were conducted in North America; 28 in the USA, seven in Canada and two from France and one from Spain. There was 26 RCTs, and 12 quasi-experimental studies. All studies included participants who experienced homelessness; 26 studies reported that participants experienced homelessness and substance use. Detailed study characteristics are in S5 File.

**Table 3:** Summary study characteristics.

Study Characteristic (n = 38)	Frequency	%
Country		
USA	28	73.6
Canada	7	18.4
Spain	1	2.6
France	2	5.2
Year		
1991–1995	5	13.1
1996–2000	5	13.1
2001–2005	5	13.1
2006–2010	3	7.8
2011–2015	6	15.7
2016–2020	9	23.6
2021+	5	13.1
Design of study		
RCT	26	68.4
Quasi-experimental	12	31.5
Outcome reported in studies		
Substance use	34	89.4
Smoking	2	5.2
Oral health	1	2.6
Diet	0	-
Mixed		
*Substance use + smoking*	1	2.6
Target population reported in studies		
Homeless	11	28.9
Homeless + Substance users	26	68.4
Homeless + Repeat offenders	0	-
Homeless + Repeat offenders + Substance users	1	2.6

### Quality appraisal of studies

**Fig 2:**
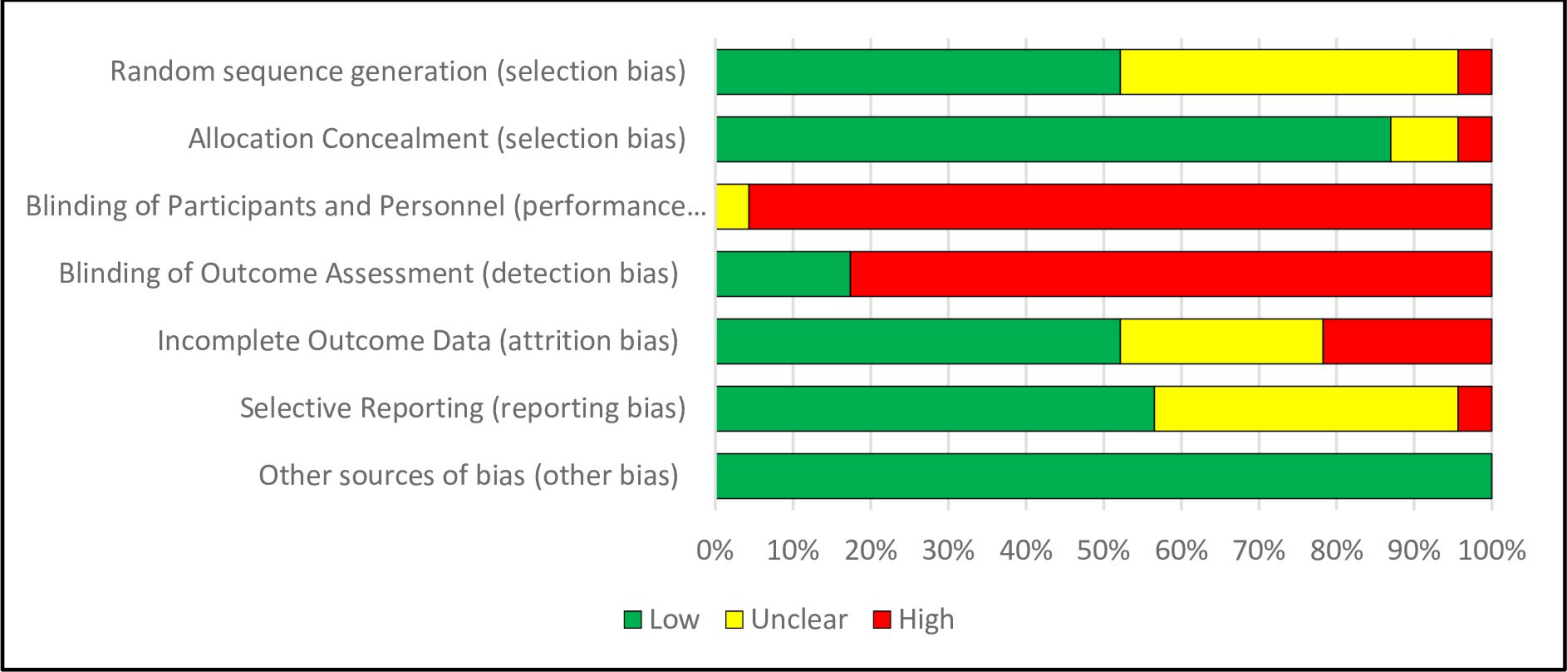
Assessments across studies for each risk of bias item.

**Fig 3:**
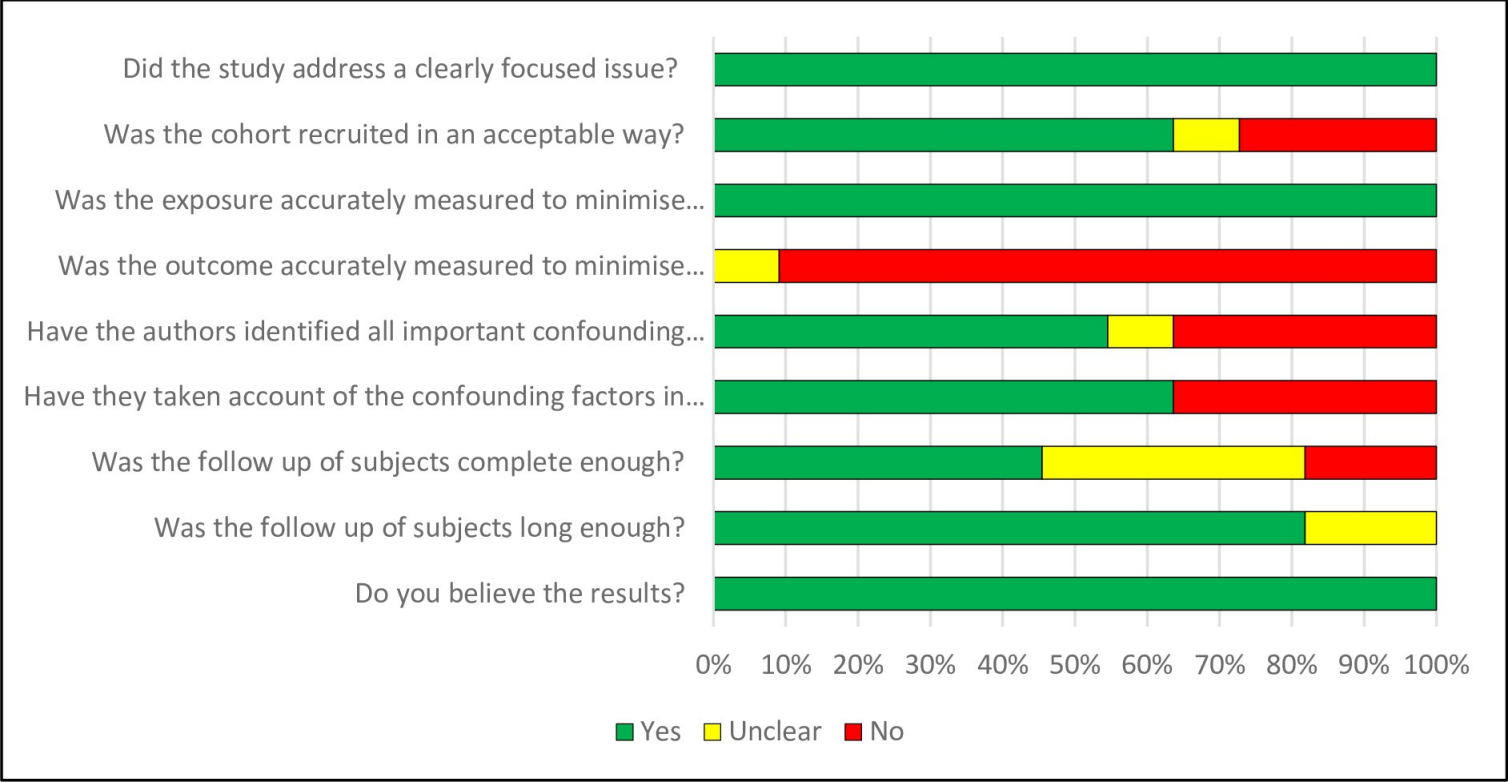
Assessments across studies for each CASP item.

For the effectiveness review, assessments across studies for each risk-of-bias item for RCTs is shown in Fig 2. Risk-of-bias assessment for each study in S6 File. Of the 23 RCTs, 91% were rated as low risk of allocation concealment [30–50]. However, 52% had high or uncertain risk of reporting bias (mostly due to use of self-report measures) [31, 34–37, 39, 41, 46–48, 51, 52] and 48% had high or uncertain risk due to attrition bias (high levels of incomplete outcome) [31, 34, 36, 37, 39, 41, 44, 47, 50–52]. A summary of the CASP Cohort Checklist for quasi-experimental studies is in Fig 3 (S7 File has CASP assessments for each study). Of the 11 quasi-experimental studies, 54% had incomplete follow-up [53–58], nearly all had some reporting bias in exposure or outcome measures, and 36% did not account for any confounders [53, 54, 56, 59].

The review on cost-effectiveness included five studies, which were over a short time horizon [55, 60–63], and two of the studies were higher quality using the BMJ checklist (S4 File) [60, 63].

### Effectiveness results

#### Primary outcomes

Most studies reported outcomes related to substance use (drugs, alcohol and combined) (n = 30). Two studies included smoking-related outcomes [45, 46], one study reported both smoking *and* substance use outcomes [51], one study reported outcomes on oral health [64]; none reported diet-related outcomes.

#### Substance use

*Summary of interventions reported*. Most studies (N = 21) comprised interventions that were multi-component, encompassing a combination of different interventions rather than one specific intervention. These were mostly substance abuse/mental health treatment integrated with housing programmes, including shelter-based treatment programs, and Housing First model (housing service with community treatment services) [50]. Other interventions centred around work-based programs, including abstinence-contingent work therapy (behavioural therapy that involves housing and employment subject to substance use abstinence), or housing with mental health/substance treatment. Interventions were categorised into structural-level (e.g. housing, work-based), community-level (e.g. assertive community treatment), and individual-level (e.g. mental health/addiction treatment) interventions. Some interventions were multi-level; these were individual/structural interventions but also included a community-level component, such as assertive community treatment, or therapeutic communities. A breakdown of intervention types by study and outcome is presented in S8 File.

*Summary of substance use outcomes reported*. Among the 30 studies reporting on substance use, outcomes were reported in three different ways: drug-specific outcomes (n = 17), alcohol-specific outcomes (n = 24), or a combined measure of substance use (i.e., both drug and alcohol-related outcomes) (n = 12). Results on these outcomes are reported below.

*Drug-specific outcomes*. Table 4 presents intervention types and overall findings (direction of effect) for studies reporting drug-related outcomes according to the three measures of addiction/severity of use, frequency of use and ‘other’ measures. An RCT with veterans experiencing homelessness and substance use reported that a compensated/abstinence-contingent work therapy (CWT) program led to reduced addiction-related problems with low attrition rates over 12 months [35]. Several studies reported reduced drug use frequency [41, 43, 52, 56]. One RCT combining day treatment (mix of group therapy and individual counselling) and work therapy showed a 36% cocaine use reduction at 2 months, not sustained at 12 months [41]. Another RCT with short-term shelter and day treatment found sustained cocaine use decrease at 21 months [52]. A behavioural therapy program for women experiencing homelessness and criminal justice involvement showed positive drug use effects at six months [43]. A two-year integrated assertive community treatment intervention aimed at reducing drug use frequency among individuals with severe mental illness experiencing homelessness [56]. However, a housing intervention study with intensive case management (Housing First Model) reported the control group had fewer drug use problems compared to the intervention group due to differential participant selection [53]. This study had differential selection of participants; participants in the comparator group had much lower health and substance use needs.

**Table 4:** Measures of outcomes, interventions, and direction of effect of for each included study reporting drug-related outcomes.

Study	Design	Measure of outcome	Brief description of intervention	Intervention Group	Findings–direction of effect (positive: green; negative: red; amber: no association)
**Addiction/severity of drug use**					
Burnam 1995	RCT	Drug use index	Social model residential treatment program (with mental health and substance use treatment)	Structural + Individual	
			Social model non-residential program (with mental health and substance use treatment)	Individual + Community	
Cherner 2017	Quasi-experimental	Drug Abuse Screening Test (DAST)-10	Housing First with intensive case management services	Structural + Individual	
Drake 1997	Quasi-experimental	Addiction severity index (ASI) Composite	Integrated treatment—mental health, substance abuse, housing	Structural + Individual	
Hwang 2011	Quasi-experimental	DAST-20	Supported housing intervention–support for housing, mental health, living skills	Structural + Individual	
Kashner 2002	RCT	ASI Composite	Department of Veterans Affairs compensated work therapy program	Individual	
Kirst 2015	RCT	ASI (days with problems)	Housing First—housing, rent supplement, and mental health, substance use	Structural + Individual	
Lam 1995	RCT	ASI Composite	Shelter-based treatment program–drug-free modified therapeutic community, group therapy, housing, employment, skill-building	Structural + Individual	
Malte 2017	RCT	ASI Composite	Intensive addiction/housing case management–case management support for housing, substance use, life skills	Community + Individual	
Mares 2011	Quasi-experimental	ASI Composite	Comprehensive housing and health care services–support for housing, primary care, mental health, substance use	Structural + Individual + Community	
Morse 2008	Quasi-experimental	Drug Use Scale	Assertive community treatment only (ACTO)	Individual + Community	
			Integrated assertive community treatment (IACT)–assertive outreach with behavioural counselling	Individual + Community	
			New integrated assertive community treatment (NIACT)–more frequent treatment sessions, more support staff	Individual + Community	
Tsai 2010	Quasi-experimental	ASI Composite	Residential treatment first before independent housing	Structural + Individual	
**Frequency of drug use/abstinence**					
Burnam 1995	RCT	ASI (past 30 days)	Social model residential treatment program (with mental health and substance use treatment)	Structural + Individual	
			Social model non-residential program	Individual + Community	
Ferreiro 2022	RCT	Not specified (binary measure of use/no use)	Housing First–permanent housing with support services and mental health care	Structural + Individual	
Lam 1995	RCT	ASI (past 30 days)	Shelter-based treatment program–drug-free modified therapeutic community, group therapy, housing, employment, skill-building	Structural + Individual	
Mares 2011	RCT	ASI (past 30 days)	Comprehensive housing and health care services–support for housing, primary care, mental health, substance use	Structural + Individual + Community	
Milby 1996	RCT	Urine toxicologies	Day treatment program + abstinent contingent work therapy + housing	Structural + Individual	
Morse 2008	Quasi-experimental	ASI (past 30 days)	Assertive community treatment only (ACTO)	Individual + Community	
			Integrated assertive community treatment (IACT)	Individual + Community	
			New integrated assertive community treatment (NIACT)–assertive outreach with behavioural counselling	Individual + Community	
Nyamathi 2017	RCT	Texas Christian University (TCU) Form II/Urine	Dialectical behavioral therapy-corrections modified program	Individual	
Stahler 1995	Quasi-experimental	ASI (past 30 days)	Integrated comprehensive residential services	Structural + Individual	
			Shelter-based intensive case management + community services	Structural + Individual	
Tsemberis 2004	RCT	Timeline followback	Housing First—immediate housing without treatment prerequisites	Structural + Individual + Community	
**Money spent on drugs**					
Kirst 2015	RCT	ASI money spent on drugs (past 30 days)	Housing First—housing, rent supplement, and mental health, substance use	Structural + Individual	
Stahler 1995	Quasi-experimental	ASI (past 30 days)	Integrated comprehensive residential services	Structural + Individual	
			Shelter-based intensive case management + community services	Structural + Individual	
**Number of different drugs/Highest frequency of drugs used**					
French 1999	Quasi-experimental	Center for Therapeutic Community Research (CTCR)	Modified therapeutic community—moderate intensity	Structural + Individual + Community	

*Alcohol-specific outcomes*. Table 5 presents findings for studies reporting alcohol-related outcomes according to measures of addiction/severity, frequency of use and ‘other measures’. Four RCTs reported a reduction in alcohol addiction-related problems. Two 3-month RCTs used non-abstinence based behavioural counselling co-developed with people with homelessness experience, resulting in decreased alcohol addiction measures [32, 33]. Combining pharmacological treatment with harm reduction counselling did not show positive effects after 12 weeks [33]. Intensive case management for people with long-standing alcohol use and homelessness reduced alcohol problems and frequency over 18 months [34]. A compensated work therapy program for veterans experiencing homelessness reduced alcohol addiction-related problems over 12 months [35]. A Housing First intervention along with support for mental health and substance use reported after 24 months a reduction in alcohol-related problems, and on money spent on alcohol, although no effect on drug-related outcomes was found [36].

**Table 5:** Measures of outcomes, interventions, and direction of effect of for each included study reporting alcohol-related outcomes.

Study	Design	Measure of outcome	Brief description of intervention	Intervention Group	Findings–direction of effect (positive: green; negative: red; amber: no association)
**Addiction/severity of alcohol use**					
Cherner 2017	Quasi-experimental	Alcohol use disorders identification test (AUDIT)	Housing First with intensive case management services	Structural + Individual	
Collins 2019	RCT	Short Inventory of Problems (SIP-2A)	Harm Reduction Treatment for Alcohol (HaRT-A)–behavioural counselling		
Collins 2021	RCT	Short Inventory of Problems (SIP-2R)	Harm Reduction Treatment for Alcohol HaRT-A + XR-NTX (behavioural counselling plus extended-release naltrexone injections	Individual	
			HaRT-A (behavioural harm reduction treatment) plus placebo injection	Individual	
			HaRT-A (behavioural counselling) only	Individual	
Cox 1998	RCT	ASI Composite	Intensive case management–long-term support for financial and residential stability, personal skills, substance use reduction	Community + Individual	
Drake 1997	Quasi-experimental	ASI Composite	Integrated treatment—mental health, substance abuse, housing	Structural + Individual	
Hwang 2011	Quasi-experimental	AUDIT	Supported housing intervention	Structural + Individual	
Kashner 2002	RCT	ASI Composite	Department of veterans affairs compensated work therapy program	Structural + Individual	
Kirst 2015	RCT	ASI problems	Housing First—housing, rent supplement, and mental health, substance use	Structural + Individual	
Lam 1995	RCT	ASI Composite	Shelter-based treatment program	Structural + Individual	
Loubière 2022	RCT	AUDIT	French Housing first—offers independent housing with assertive community therapy	Structural +Individual + Community	
Malte 2017	RCT	ASI Composite	Intensive addiction/housing case management—individualized housing, substance use and mental health case management, life skills training, and community outreach	Structural + Individual	
Mares 2011	Quasi-experimental	ASI Composite	Comprehensive housing and health care services	Structural + Individual + Community	
Morse 2008	Quasi-experimental	Drug Use Scale	Assertive community treatment only (ACTO)	Individual + Community	
			Integrated assertive community treatment (IACT)	Individual + Community	
			New integrated assertive community treatment (NIACT)	Individual + Community	
Orwin 1994 [72]	Quasi-experimental	ASI Composite	Boston: Outreach, residential recovery, housing, case mx, transport	Structural + Individual	
			Los Angeles: Residential recovery, transport	Structural + Individual	
			Louisville: Outreach, shelter, drug treatment, case mx, transport	Structural + Individual	
			Minneapolis: Case mx, transport	Individual	
			New York: Outreach, non-residential recovery, case mx, transport	Individual	
Stockwell 2021	Quasi-experimental	AUDIT	Managed Alcohol Programs–housing, harm reduction and health care services	Structural +Individual	
		Severity of Alcohol Dependence Questionnaire (SADQ)		Structural +Individual	
Tsai 2010	Quasi-experimental	ASI Composite	Residential treatment first	Structural + Individual	
**Frequency of alcohol use/abstinence**					
Burnam 1995	RCT	ASI (past 30 days)	Social model residential treatment program	Structural + Individual	
			Social model non-residential program	Individual + Community	
Collins 2019	RCT	ASI (past 30 days)	HaRT-A—behavioural harm reduction treatment sessions	Individual	
Collins 2021	RCT	ASI (past 30 days)	HaRT-A + XR-NTX—behavioural harm reduction treatment + intramuscular injections of extended-release naltrexone	Individual	
			HaRT-A—behavioural harm reduction treatment + placebo injection	Individual	
			HaRT-A—behavioural harm reduction treatment only	Individual	
Cox 1998	RCT	ASI (past 30 days)	Intensive case management—housing, financial, substance use reduction	Structural + Individual	
Ferreiro 2022	RCT	Not specified (binary measure of use/no use)	Housing First–permanent housing with support services and mental health care	Structural + Individual	
French 1999	Quasi-experimental	CTCR	Modified therapeutic community—moderate intensity	Structural + Individual + Community	
Koffarnus 2011	RCT	Breath samples	Therapeutic Workplace: paid job skills training (abstinent contingent)	Structural + Individual	
			Therapeutic Workplace: paid job skills training (no abstinence contingencies)	Structural + Individual	
Lam 1995	RCT	ASI (past 30 days)	Shelter-based treatment program	Structural + Individual	
Mares 2011	RCT	ASI (past 30 days)	Comprehensive housing and health care services	Structural + Individual + Community	
Milby 1996	RCT	ASI (past 30 days)	Day treatment program + abstinent contingent work therapy + housing	Structural + Individual	
Morse 2008	Quasi-experimental	ASI (past 30 days)	Assertive community treatment only (ACTO)	Individual + Community	
			Integrated assertive community treatment (IACT)	Individual + Community	
			New integrated assertive community treatment (NIACT)	Individual + Community	
Nyamathi 2017	RCT	TCU Form II/Urine	Dialectical behavioral therapy-corrections modified program	Individual	
Orwin 1994 [72]	Quasi-experimental	ASI (past 30 days) and abstinence	Boston: Outreach, residential recovery, housing, case mx, transport	Structural + Individual	
			Los Angeles: Residential recovery, transport	Structural + Individual	
			Louisville: Outreach, shelter, drug treatment, case mx, transport	Structural + Individual	
			Minneapolis: Case mx, transport	Individual	
			New York: Outreach, non-residential recovery, case mx, transport	Individual	
Stahler 1995	Quasi-experimental	ASI (past 30 day use)	Integrated comprehensive residential services	Structural + Individual	
			Shelter-based intensive case management + community services	Structural + Individual	
Tsemberis 2004	RCT	Time Line Follow Back	Housing First—immediate housing without treatment prerequisites	Structural + Individual + Community	
**Money spent on alcohol**					
Kirst 2015	RCT	ASI (past 30 days)	Housing First—housing, rent supplement, and mental health, substance use	Structural + Individual	
Stahler 1995	Quasi-experimental	ASI (past 30 days)	Integrated comprehensive residential services	Structural + Individual	
			Shelter-based intensive case management + community services		
**Peak alcohol quantity**					
Collins 2019	RCT	Alcohol Quantity Use Assessment	HaRT-A—behavioural harm reduction treatment sessions	Individual	
Collins 2021	RCT	Alcohol Quantity Use Assessment	HaRT-A + XR-NTX—behavioural harm reduction treatment + intramuscular injections of extended-release naltrexone	Individual	
			HaRT-A—behavioural harm reduction treatment + placebo injection	Individual	
			HaRT-A—behavioural harm reduction treatment only	Individual	

In terms of frequency of alcohol use, six studies reported a positive result. Two were RCTs on behavioural counselling [32, 33], and one involving intensive case management [34], which also showed positive effects on addiction/ severity of alcohol use. A 4 –year therapeutic workplace contingent programme showed some evidence of a positive impact, although there was greater under-reporting in the control group [37]. Two RCTs, one based on a 12 -month enhanced day treatment programme plus abstinent-contingent work therapy and housing [41], and the other with a 3-month behavioural therapy programme for women experiencing homelessness who also had involvement with the criminal justice system reported beneficial effects on reducing alcohol use at the 6-month follow up [43]. Both these studies also reported positive effects on reducing drug use (Table 4). The two RCTs on behavioural counselling also showed a reduction in peak alcohol consumption [32, 33]. Cherner [53], (similar to their findings on drug-related outcomes in Table 4), reported fewer problems with alcohol use in the control compared to intervention group, although alcohol related problems decreased in both groups at 24 months. Participants in the comparator group had much lower health and substance use needs.

*Combined substance use outcomes*. Amongst studies reporting substance use combined (drugs and alcohol) (Table 6), one RCT examining Housing First interventions, including mental health support, showed a reduction in addiction/severity of substance use, which was significant at 12 months and not at 24 months [36]. This intervention also reported a positive impact on alcohol-related addiction (Table 5). Two RCTs at 6 months and 12 months respectively found that housing with day treatment was effective in reducing frequency of substance use [40, 42], with abstinence-contingent housing having a more positive effect on substance use [42]. Quasi-experimental studies found that case management was associated with a reduction in drug use [57], and integrated treatment (mental health, substance use and housing) resulted in greater recovery from substance use [54].

**Table 6:** Measures of outcomes, interventions, and direction of effect of for each included study reporting combined substance use outcomes.

Study	Design	Measure of outcome	Brief description of intervention	Intervention Group	Findings–direction of effect (positive: green; negative: red; amber: no association)
**Addiction/severity of substance use**					
Aubry 2019	RCT	Global Appraisal of Individual Needs–Short Screener (GAIN-SS)	Housing First + assertive community treatment	Structural + Individual + Community	
Kirst 2015	RCT	GAIN-SS	Housing First—housing, rent supplement, and mental health	Structural + Individual	
O’Campo 2016	RCT	GAIN-SS	Housing First + assertive community treatment	Structural + Individual + Community	
Loubière 2022	RCT	Section K of the Mini International Neuropsychiatric Interview (MINI)	French Housing First (HF)—Independent housing with Assertive community therapy (ACT)	Structural+ Individual + Community	
**Frequency of substance use/abstinence**					
Malte 2017	RCT	ASI (past 30 days)	Intensive addiction/housing case management	Structural + Individual	
Milby 2000	RCT	Urine samples	Behavioral day treatment + abstinence contingent housing and work therapy	Structural + Individual	
Milby 2005	RCT	Urine samples	Day treatment + housing contingent on drug abstinence	Structural + Individual	
			Day treatment + housing not contingent on abstinence	Structural + Individual	
Nyamathi 2017	RCT	TCU Form II/Urine	Dialectical behavioral therapy-corrections modified program	Individual	
Somers 2015a	RCT	Maudsley Addiction Profile	Housing First with intensive case management	Structural + Individual	
Somers 2015b	RCT	Maudsley Addiction Profile	Housing First (scattered site) with assertive community treatment	Structural + Individual + Community(?)	
			Congregate housing (single building) with on-site 24/7 support	Structural + Individual	
Sosin 1995	Quasi-experimental	No specific measure given (ASI??)	Case management only	Individual	
			Case management + supported housing	Structural + Individual	
Slesnick 2023	RCT	Form 90	Housing + supportive services	Structural + Individual	
			Housing only	Structural	
**Treatment progression**					
Drake 1997	Quasi-experimental	Substance Abuse Treatment Scale (SATS)	Integrated treatment—mental health, substance abuse, housing	Structural + Individual	

The remaining studies did not report significant effects or reductions across substance use outcomes [30, 31, 37–39, 44, 48–52, 54, 55, 58, 59, 65–67] Most of these studies had high attrition, self-reported measures, low adherence to interventions, and differential health problems and attrition rates in the intervention and comparison groups.

#### Meta-analysis results

**Fig 4:**
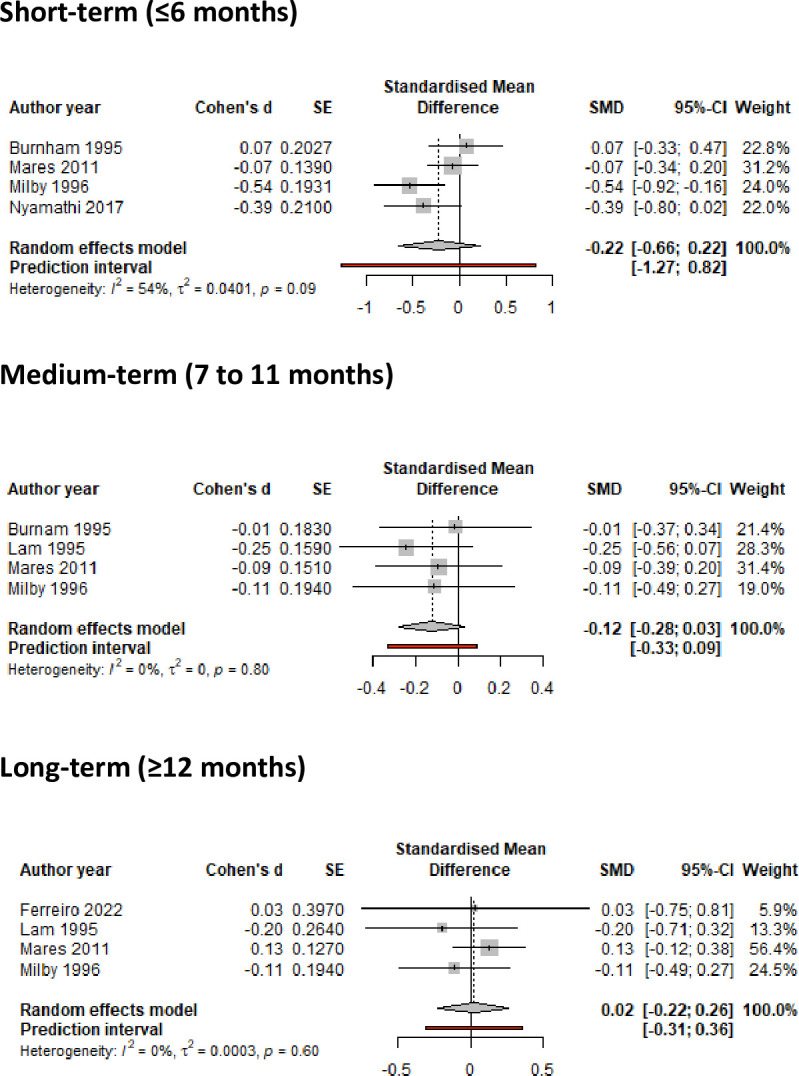
Meta-analysis of studies reporting frequency of drug use in the short-, medium- and long-term.

**Fig 5:**
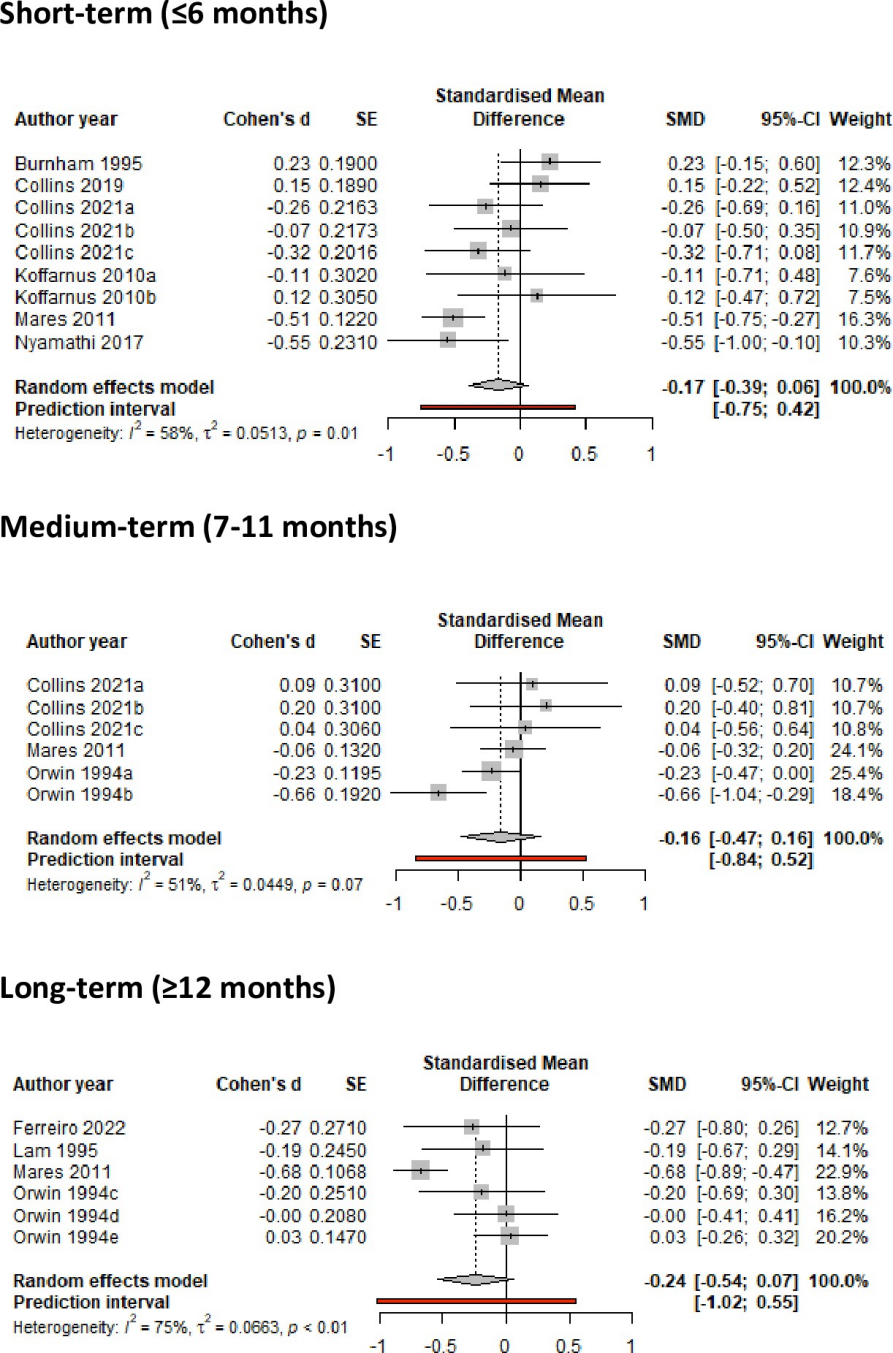
Meta-analysis of studies reporting frequency of alcohol use in the short-, medium- and long-term.

**Fig 6:**
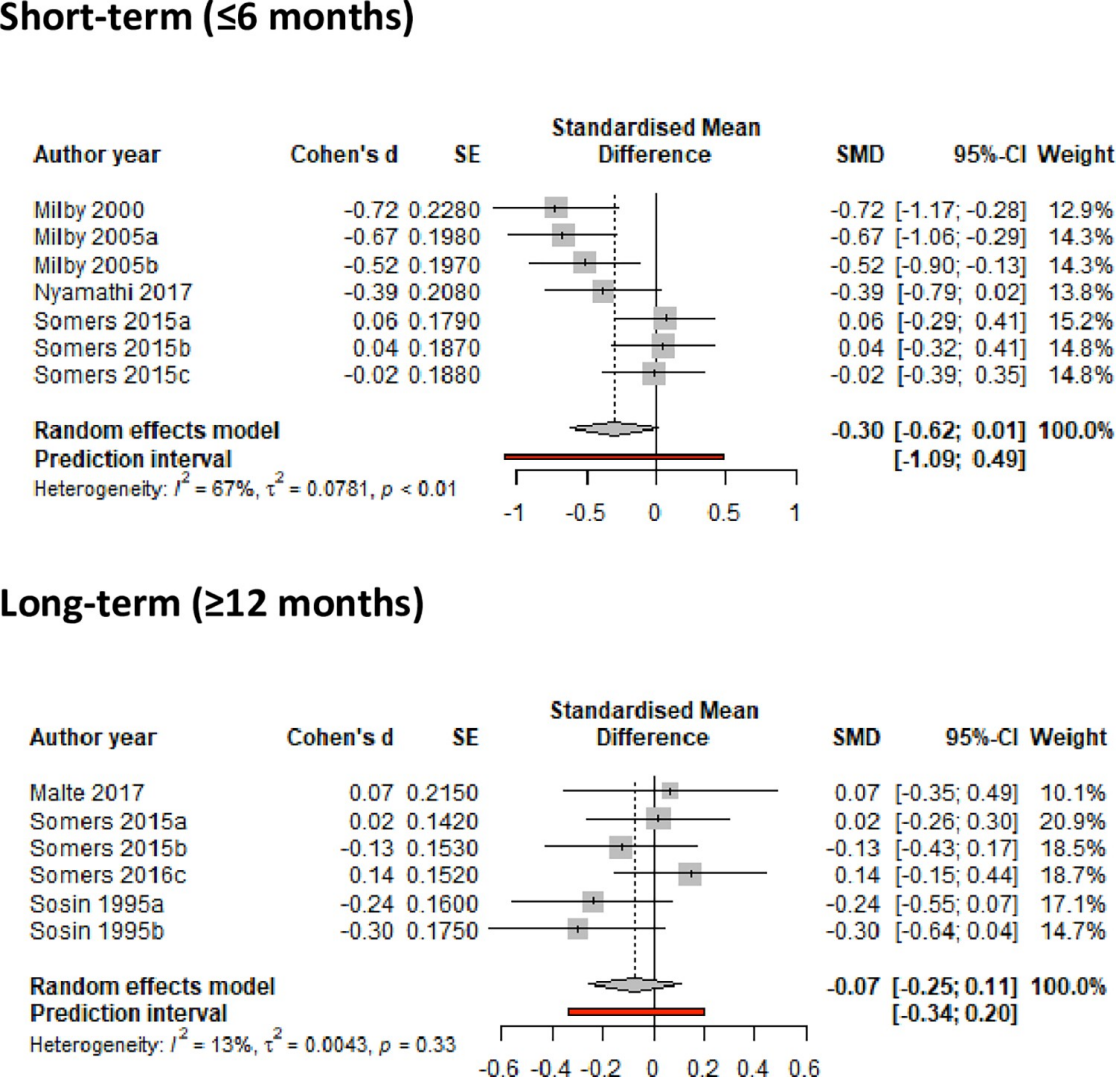
Meta-analysis of studies reporting frequency of combined drug and alcohol use in the short- and long-term.

Meta-analysis showed no statistically significant reductions in any of the outcomes related to frequency of substance use, although interventions led to some (albeit non-significant) reduction in frequency of drug use (or increase in abstinence) in the medium-term (SMD = -0.12 (95%CI -0.28, 0.03), but not for long-term reduction (Fig 4). Meta-analysis of studies reporting frequency of alcohol use showed no significant evidence of a positive effect in the short-term (SMD = -0.17 95%CI -0.39, 0.06) and long-term (SMD = -0.24; 95%CI -0.54, 0.07) (Fig 5). A small positive, although non-significant, effect was observed for reduction in frequency of combined drug use (SMD = -0.30; 95%CI -0.62, 0.01) (Fig 6). The meta-analysis evidence appeared to be mostly of low certainty by GRADE assessments due to heterogeneity of interventions, wide confidence intervals and high risk of bias.

#### Smoking

Three RCTs reported outcomes related to smoking or tobacco use (Table 7) [45, 46, 51]. An RCT reported that nicotine replacement with contingency management prize in addition to standard nicotine replacement and counselling achieved higher abstinence and self- reported 4-week quit rate, however, there was no difference in the groups after a 6-month follow-up [46]. Studies examining the Housing First intervention, or motivational interviewing along with nicotine replacement did not find significant differences in smoking levels compared to standard care [45, 51].

**Table 7:** Measures of outcomes, interventions, and direction of effect of for each included study reporting smoking-related outcomes.

Study	Design	Measure of outcome	Brief description of intervention	Intervention Group	Findings–direction of effect (positive: green; negative: red; amber: no association)
Okuyemi 2013	RCT	Self-reported abstinence + carbon monoxide (CO) samples	Nicotine patch + motivational interviewing	Individual	
Rash 2018	RCT	Self-reported abstinence + CO samples	Nicotine replacement therapy, counselling, CO monitoring + contingency management	Individual	
Ferreiro 2022	RCT	Tobacco use	Housing First–housing provision and supporting services, incl. mental health	Structural + Individual	

#### Oral health

One quasi-experimental study in the USA reported oral health related outcomes, including access to a dental specialist and tooth decay [64]. The intervention comprised provision of medical, dental, and social services and health education to residents within transitional housing facilities. The study found the intervention did not result in improving in access to dental care or tooth decay levels at 6 or 18 months follow-up when compared to standard care.

### Secondary outcomes

Secondary outcomes, including mental health, quality of life, housing, employment, and income, were reported in some studies. Housing related outcomes were most reported, with studies reporting positive housing-related effects. Improvements in quality of life were reported in six studies [30, 33, 38, 51, 54, 65], and improvements in mental health [51, 54, 55], and income [34, 52, 66] were reported in three studies each.

### Cost-effectiveness results

Five studies reported on economic evaluations of interventions, which showed an improvement in substance use [55, 60–63]. The intervention in these studies were modified therapeutic community for those experiencing homelessness and mental illness [55, 60], enhanced care programs [62, 63], and Housing First [61]. All studies found that interventions were cost-effective (in comparison with usual care) for abstinence in SMD groups; different analytical measures were reported in the studies, including cost-effectiveness ratio, cost-benefit ratio and cost and outcome ratios. Some caution should be applied in these results as the time horizon of studies is limited to capture all possible cost and effectiveness outcomes of interventions.

## Discussion

This review focused on populations experiencing severe and multiple disadvantage (SMD) and assessed the effectiveness and cost-effectiveness of interventions to improve oral health and the related behaviours of substance use, smoking and diet. Thirty studies on effectiveness of interventions reported on substance use, three studies reported on smoking, one on oral health related outcomes, and none on diet. Studies reporting substance use outcomes comprised interventions which were mostly multi-component and acting at individual, community, and/or structural levels. Studies suggested some (albeit weak) evidence for reduction in substance use were related to work therapy and housing (both abstinence-contingent and non-abstinence contingent), intensive case management; and integrated programmes combining substance use and mental health support. Two studies reported beneficial effects of individual-level programmes (behavioural counselling).

Overall, several types of interventions in the included studies reported impacts on substance use in SMD groups. Interventions ranged from housing-based programmes, enhanced community-based support and behavioural counselling. These interventions included ones that aimed at supporting housing, along with work-based therapy, housing integrated with mental health and substance use support, and a combination of intensive counselling and behavioural therapy, housing support, rent and life-skills support. In addition, for alcohol use reductions, harm reduction interventions appeared to show some positive results. Although some studies showed reductions in substance use, most of the effects were weak and non-significant. Limited evidence showed that interventions were cost-effective in reducing substance use outcomes. These findings align (in terms of direction of effects) with a recent review that also found that harm-reduction and case management approaches reduce substance consumption in people experiencing SMD [10]. Our review had a broader scope in terms of focusing on homelessness and substance use as well as offending. Input from policy and practice stakeholders and people experiencing homelessness on the review findings suggested the need to assess substance use, separately for drug and alcohol use. Our review found that far more studies reported on alcohol-related issues (N = 24) than drug use, and fewer reported on use of both alcohol and drugs.

A notable gap in evidence identified in this review is the lack of evidence on effectiveness of interventions for improving oral health and diet among SMD groups. Poor oral health has significant impacts on the health and well-being of people experiencing SMD [8]. Whilst there are several studies reporting on challenges of oral health in this population and issues related to barriers accessing care [5, 68–70], there remains a need for intervention studies to assess and report on oral health outcomes. Interventions related to reducing substance use are important for improving oral health outcomes because of the detrimental impacts of substance use on oral health as evidence has shown that the use of drugs such as opiates, cocaine and methamphetamines result in increased levels of tooth day, tooth wear and periodontitis [71]. Yet, only one study reported oral health measures and found no difference in oral health outcomes such as access to dental care and tooth decay levels [64]. There was also limited reporting on whether interventions were co-produced or developed with peer groups or people with lived experience, a particular gap given recent NICE guidance encouraging co-produced interventions [12].

The review has many strengths. This systematic review adds to the current evidence as it compiles evidence on the effectiveness and cost-effectiveness of interventions specifically targeting oral health and related health behaviours in SMD groups. Methodological rigour was applied to ensure that screening, data extraction, and quality assessments were done in duplicate or checked in full by a second reviewer to minimise error. By focusing on SMD populations, the review takes account of the related aspects of disadvantage faced by people experiencing homelessness. An approach recognising the role of whole systems and wider determinants of health was adopted, such that interventions of all types were included; this allowed us to assess interventions acting at structural, community and individual levels. The review was also strengthened by input from people with lived experience of SMD and policy and practice stakeholders; for example, relevant outcomes where identified based on their perspectives through a series of remote and in-person workshops. This guided us in initially refining our search strategy and eventually conceptualising outcomes related to substance use, in terms of both frequency of use and dependence (or addiction).

The quality of evidence found in this review was affected by some limitations which were common to several studies. Most studies had small sample sizes, and short durations of follow-up. Levels of attrition of the sample were also high, with difficulties in retention of participants over the course of studies. Although some studies utilised more objective measures of substance use, others had self-reported measures increasing the chances of reporting bias. Further, most included studies were based in the USA and Canada, with limited evidence from other countries. The degree to which findings could be synthesised to reach firm conclusions were constrained by heterogeneity between studies regarding intervention design (for example, single- or multi-component), outcomes (oral health, alcohol, drug, smoking use and diet), and evaluation approach (for example, study design; measurements utilised). Meta-analysis undertaken in our review had high levels of heterogeneity and the findings should therefore be interpreted with caution.

### Implications of findings

The review suggested some, albeit weak, evidence that interventions targeting different aspects of disadvantage have the potential to reduce substance use in people with SMD. The evidence was largely from studies that combined interventions, such as housing support, mental health, work-based support, behavioural counselling, enhanced support, or case management. This approach recognises the multiple and co-occurring needs of people experiencing homelessness, which was also highlighted in the NICE guidance on integrated care [12]. Further research from high quality studies is needed, in particular studies that are adequately powered, have greater retention and are able to assess the longer-term impacts of interventions on these health outcomes. Issues of attrition are challenging in studies with SMD populations facing transient and difficult circumstances. Adopting stronger engagement with people with lived experience and frontline services supporting these populations are considerations for future studies. The review also highlights the need for further research related to oral health and diet related in evaluation studies in SMD groups.

## Supporting information

S1 Checklist PRISMA checklist. (DOCX)

S1 File Methods. Initial Logic Model.
(DOCX)

S2 File Methods. Search Strategy.
(DOCX)

S3 File Table A. GRADE Assessment. (DOCX)

S4 File Table B. Quality assessment of Economic Evaluation Studies; Drummond BMJ checklist. (DOCX)

S5 File Table C. Detailed study characteristics of included studies. (DOCX)

S6 File Fig A. Risk of bias across each domain for each included study. (DOCX)

S7 File Table D. CASP assessments for each included study. (DOCX)

S8 File Table E. Detailed intervention breakdown by study and outcome type. (DOCX)
