# Supplementary figures and images for: Improving oral health and related health behaviours (substance use, smoking, diet) in people with severe and multiple disadvantage: A systematic review of effectiveness and cost-effectiveness of interventions
Source: PLoS One. 2024 Apr 18;19(4):e0298885. doi: 10.1371/journal.pone.0298885 (PMC11025870; doi:10.1371/journal.pone.0298885)

# **Methods. Initial Logic Model**


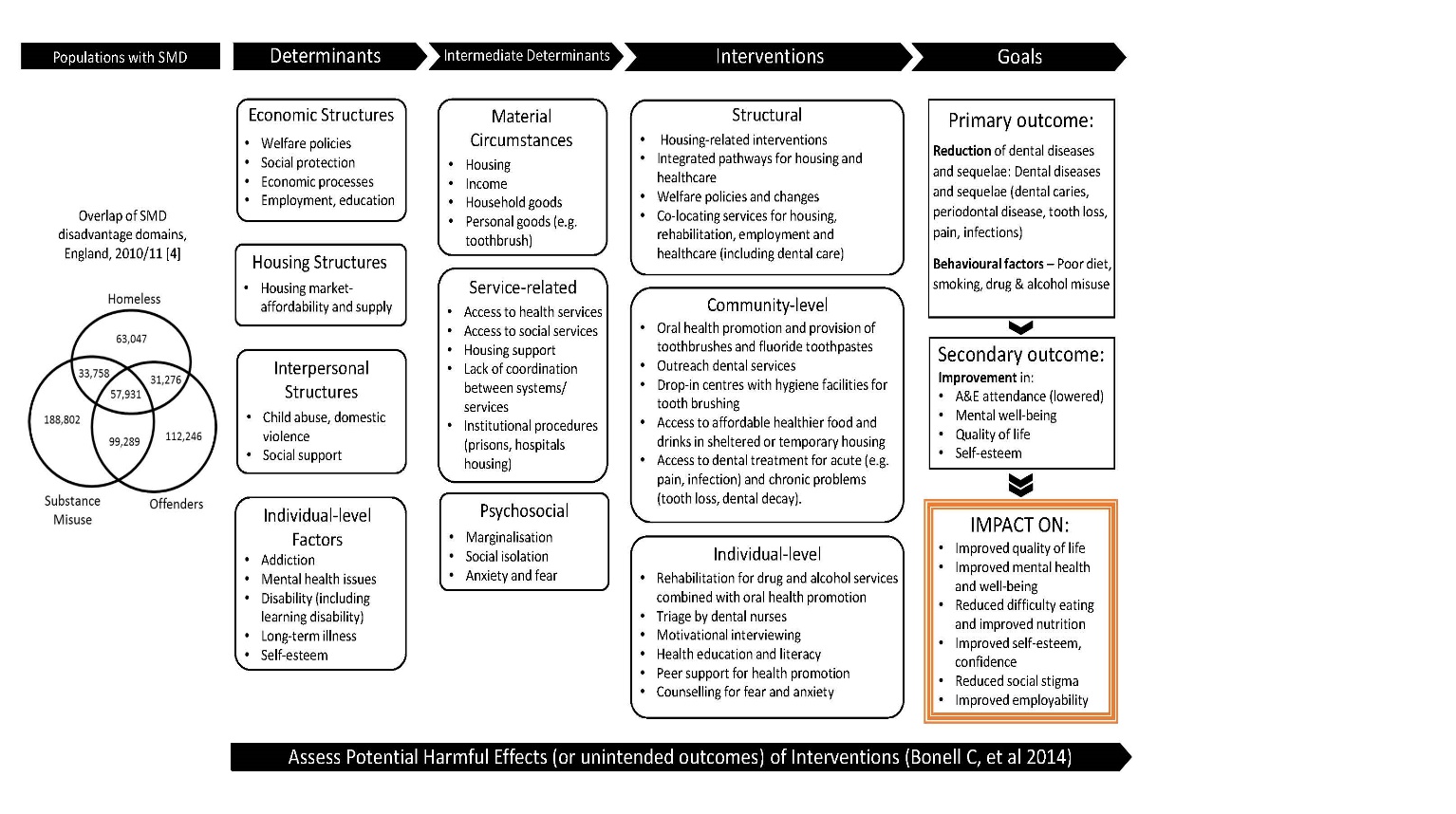

Supplement: S1 File — Initial Logic Model. (DOCX) [file pone.0298885.s002.docx]
