# Supplementary material for: Improving oral health and related health behaviours (substance use, smoking, diet) in people with severe and multiple disadvantage: A systematic review of effectiveness and cost-effectiveness of interventions
Source: PLoS One. 2024 Apr 18;19(4):e0298885. doi: 10.1371/journal.pone.0298885 (PMC11025870; doi:10.1371/journal.pone.0298885)
Supplement: S2 File — Search Strategy. (DOCX) [file pone.0298885.s003.docx]

# **Methods. Search Strategy**

| **#** | **Searches** |
| --- | --- |
| 1 | Homeless Persons/ |
| 2 | homeless*.ti,ab,kw,kf. |
| 3 | ((hous* or home* or accommodat* or shelter) adj3 (insecur* or instability or unstable or stability)).ti,ab,kw,kf. |
| 4 | (sleep* adj2 rough).ti,ab,kw,kf. |
| 5 | squatter*.ti,ab,kw,kf. |
| 6 | shelter.ti,ab,kw,kf. |
| 7 | "sofa surf*".ti,ab,kw,kf. |
| 8 | or/1-7 |
| 9 | ((severe or multiple) adj disadvantage*).ti,ab,kw,kf. |
| 10 | "social exclusion".ti,ab,kw,kf. |
| 11 | "complex needs".ti,ab,kw,kf. |
| 12 | "marginali?ed populations".ti,ab,kw,kf. |
| 13 | or/9-12 |
| 14 | exp Substance-Related Disorders/ |
| 15 | Behavior, Addictive/ |
| 16 | addict*.ti,ab,kw,kf. |
| 17 | ((alcohol or drug or substance) adj1 (misuse or abuse or use* or addict* or dependenc* or issue* or problem)).ti,ab,kw,kf. |
| 18 | Alcoholics/ |
| 19 | alcoholic*.ti,ab,kw,kf. |
| 20 | (drug adj1 (habit or tak* or hard or illicit or inject*)).ti,ab,kw,kf. |
| 21 | exp Illicit Drugs/ |
| 22 | Alcohol Drinking/ |
| 23 | "street drink*".ti,ab,kw,kf. |
| 24 | or/14-23 |
| 25 | Prisoners/ |
| 26 | prisoner*.ti,ab,kw,kf. |
| 27 | Criminals/ |
| 28 | criminal*.ti,ab,kw,kf. |
| 29 | ((repeat or ex or re or revolving door) adj1 offen*).ti,ab,kw,kf. |
| 30 | (convict or convicts or "convicted person*").ti,ab,kw,kf. |
| 31 | or/25-30 |
| 32 | 8 or 13 or 24 or 31 |
| 33 | Oral Health/ |
| 34 | ("oral health" or "dental health").ti,ab,kw,kf. |
| 35 | Oral Hygiene/ |
| 36 | "oral hygiene".ti,ab,kw,kf. |
| 37 | Mouth Rehabilitation/ |
| 38 | "mouth rehabilitation*".ti,ab,kw,kf. |
| 39 | Dental Health Services/ |
| 40 | "dental health service*".ti,ab,kw,kf. |
| 41 | Dental Care/ |
| 42 | "dental care".ti,ab,kw,kf. |
| 43 | exp Dental Caries/ |
| 44 | "dental caries".ti,ab,kw,kf. |
| 45 | Dental Enamel Solubility/ |
| 46 | "dental enamel solubility".ti,ab,kw,kf. |
| 47 | Dental Deposits/ |
| 48 | "dental deposits".ti,ab,kw,kf. |
| 49 | Dentin Sensitivity/ |
| 50 | "dentin sensitivity".ti,ab,kw,kf. |
| 51 | Dental Plaque/ |
| 52 | "dental plaque".ti,ab,kw,kf. |
| 53 | exp Dental Pulp Diseases/ |
| 54 | "dental pulp disease*".ti,ab,kw,kf. |
| 55 | Tooth Loss/ |
| 56 | "tooth loss".ti,ab,kw,kf. |
| 57 | "loss of teeth".ti,ab,kw,kf. |
| 58 | Tooth Diseases/ |
| 59 | ("tooth disease" or "diseased teeth").ti,ab,kw,kf. |
| 60 | Toothache/ |
| 61 | (toothache or "tooth ache").ti,ab,kw,kf. |
| 62 | Tooth Demineralization/ |
| 63 | Tooth Mobility/ |
| 64 | Tooth Discoloration/ |
| 65 | (tooth adj1 (demineralization or mobility or decay or discolo?ration)).ti,ab,kw,kf. |
| 66 | Mouth Diseases/ |
| 67 | Periodontal Diseases/ |
| 68 | Gingival Diseases/ |
| 69 | ((mouth or peridontal or gingival or gum) adj1 disease*).ti,ab,kw,kf. |
| 70 | exp Periodontitis/ |
| 71 | periodontitis.ti,ab,kw,kf. |
| 72 | Preventive Dentistry/ |
| 73 | "Preventive Dentistry".ti,ab,kw,kf. |
| 74 | exp Gingivitis/ |
| 75 | ((oral or dental or tooth or teeth) adj1 abscess).ti,ab,kw,kf. |
| 76 | (dental adj1 (pain or sequelae)).ti,ab,kw,kf. |
| 77 | "bleeding gum*".ti,ab,kw,kf. |
| 78 | (hole* adj2 (tooth or teeth)).ti,ab,kw,kf. |
| 79 | or/33-78 |
| 80 | exp Mouth Neoplasms/ |
| 81 | ((oral or mouth or tongue or salivary or parotid) adj1 (cancer* or tumour* or tumor* or neoplasm*)).ti,ab,kw,kf. |
| 82 | 80 or 81 |
| 83 | 79 or 82 |
| 84 | exp Smoking/ |
| 85 | (smoking or smoker or smoke or smoked or smokes).ti,ab,kw,kf. |
| 86 | Tobacco/ |
| 87 | "Tobacco Use Cessation"/ or exp "Tobacco Use"/ or exp "Tobacco Use Cessation Devices"/ |
| 88 | Smoking Cessation/ |
| 89 | "smoking cessation*".ti,ab,kw,kf. |
| 90 | Tobacco Products/ |
| 91 | Nicotine/ |
| 92 | (tobacco or cigar* or e-cig* or nicotine or hookah or pipe or vaping or vape).ti,ab,kw,kf. |
| 93 | or/84-92 |
| 94 | ((sugar or sucrose or fructose or glucose) adj2 (intake or consum*)).ti,ab,kw,kf. |
| 95 | "high sugar diet".ti,ab,kw,kf. |
| 96 | "sugary foods".ti,ab,kw,kf. |
| 97 | exp Dietary Sugars/ |
| 98 | ((processed or acidic) adj1 food*).ti,ab,kw,kf. |
| 99 | ((sugary or fizzy or carbonated or soft) adj1 drink*).ti,ab,kw,kf. |
| 100 | carbonated beverages/ or sugar-sweetened beverages/ |
| 101 | soda.ti,ab,kw,kf. |
| 102 | or/94-101 |
| 103 | ((abuse or sniff*) adj3 (solvent* or glue or gas or aerosol* or inhalant)).ti,ab,kw,kf. |
| 104 | addict*.ti,ab,kw,kf. |
| 105 | ((alcohol or drug or substance) adj1 (misuse or abuse or use* or addict* or dependen* or issue* or problem or prevention or treatment or recovery or  habit)).ti,ab,kw,kf. |
| 106 | alcoholic*.ti,ab,kw,kf. |
| 107 | Alcoholics/ |
| 108 | (drug adj1 (tak* or hard or illicit or inject*)).ti,ab,kw,kf. |
| 109 | exp Illicit Drugs/ |
| 110 | "street drink*".ti,ab,kw,kf. |
| 111 | or/103-110 |
| 112 | 83 or 93 or 102 or 111 |
| 113 | 32 and 112 |
| 114 | intervention*.ti,ab,kw. |
| 115 | program*.ti,ab,kw. |
| 116 | (care adj3 package*).ti,ab,kw. |
| 117 | feasab*.ti,ab,kw. |
| 118 | acceptab*.ti,ab,kw. |
| 119 | efficacy.ti,ab,kw. |
| 120 | effective*.ti,ab,kw. |
| 121 | training.ti,ab,kw. |
| 122 | educat*.ti,ab,kw. |
| 123 | evaluat*.ti,ab,kw. |
| 124 | strateg*.ti,ab,kw. |
| 125 | pilot.ti,ab,kw. |
| 126 | perception*.ti,ab,kw,kf. |
| 127 | belief*.ti,ab,kw,kf. |
| 128 | uptake.ti,ab,kw,kf. |
| 129 | impact.ti,ab,kw,kf. |
| 130 | consequence*.ti,ab,kw,kf. |
| 131 | attitude*.ti,ab,kw,kf. |
| 132 | barrier*.ti,ab,kw,kf. |
| 133 | facilitat*.ti,ab,kw,kf. |
| 134 | motivat*.ti,ab,kw,kf. |
| 135 | or/114-134 |
| 136 | 113 and 135 |
| 137 | limit 136 to english language |
