# Supplementary material for: Improving oral health and related health behaviours (substance use, smoking, diet) in people with severe and multiple disadvantage: A systematic review of effectiveness and cost-effectiveness of interventions
Source: PLoS One. 2024 Apr 18;19(4):e0298885. doi: 10.1371/journal.pone.0298885 (PMC11025870; doi:10.1371/journal.pone.0298885)
Supplement: S3 File — (DOCX) [file pone.0298885.s004.docx]

# **Table A. GRADE Assessment**

| **OUTCOMES** | **RISK OF BIAS (ROB)** | **INCONSISTENCY** | **INDIRECTNESS** | **IMPRECISION** | **PUBLICATION BIAS** | **GRADE** |
| --- | --- | --- | --- | --- | --- | --- |
| Alcohol – long term | V. serious (3 studies included in this outcome, 2 have high ROB and one is uncertain.) | serious (I^2^= 57% - average heterogeneity, CI don’t overlap.) | Not serious (Similar populations and interventions) | Serious (Wide CI, sample size is relatively large) | N/A (unable to assess due to low study numbers) | low |
| Alcohol – medium term | Serious (one – high ROB, one – low ROB) | Not serious (I^2^ = 0% - homogeneity but CI don’t overlap and p value = 0.07) | Serious (different interventions) | Serious (Wide CI, sample size is relatively large) | N/A (unable to assess due to low study numbers) | low |
| Alcohol- short term | Serious (4 – uncertain, one – High and one – low ROB) | Serious (I^2^=58% - average heterogeneity, CI’s don’t overlap, p=0.01, τ^2^= 0.0513 – moderate heterogeneity, PI = (-0.75, 0.42)) | Serious (different comparators and interventions) | Not serious (narrow CI, relatively decent sample size) | N/A (unable to assess due to low study numbers) | low |
| Drugs – long term | Serious (2 – high ROB, 2- uncertain ROB) | Not serious (I^2^=0%, p value = 0.60, τ^2^ = 0.0003 indicates homogeneity) | Not serious (similar populations and interventions) | Serious (wide CI) | N/A (unable to assess due to low study numbers) | low |
| Drugs – medium term | Serious (2 – high ROB, 2- uncertain ROB) | Not serious (I^2^=0%, p value = 0.80, τ^2^ = 0 indicates homogeneity) | Not serious (similar populations and interventions) | Not serious (narrow CI) | N/A (unable to assess due to low study numbers) | moderate |
| Drugs – short term | Serious (2 – high ROB, 2- uncertain ROB) | Serious (I^2^= 54%, p value = 0.08, τ^2^ = 0.0401 leaning towards moderate heterogeneity) | Serious (slight differences in intervention) | serious (wide CI, decent sample size) | N/A (unable to assess due to low study numbers) | low |
| Combined – long term | Serious (1 – high ROB, 1 – low ROB) | Not serious (I^2^= 0%, CI (-0.17, 0.21), indicates homogeneity | Not serious (slight differences in population) | Not serious (narrow CI) | N/A (unable to assess due to low study numbers) | Moderate |
| Combined – short term | Serious (2 – low ROB, 2 – uncertain ROB) | Serious (I^2^=67%, CI don’t overlap, τ^2^ = 0.0781, p <0.01) | Not serious (slight differences in interventions) | Serious (wide CI, moderate sample size) | N/A (unable to assess due to low study numbers) | low |
