# Supplementary material for: Improving oral health and related health behaviours (substance use, smoking, diet) in people with severe and multiple disadvantage: A systematic review of effectiveness and cost-effectiveness of interventions
Source: PLoS One. 2024 Apr 18;19(4):e0298885. doi: 10.1371/journal.pone.0298885 (PMC11025870; doi:10.1371/journal.pone.0298885)
Supplement: S4 File — (DOCX) [file pone.0298885.s005.docx]

# **Table B. Quality assessment of Economic Evaluation Studies; Drummond BMJ checklist**

| **1=Yes, 2= Not applicable, 3=Unclear, 4=No** | **French 1999** | **French 2002** | **Schumacher 2002** | **Mennemeyer2017** | **Tinland 2020** |
| --- | --- | --- | --- | --- | --- |
| 1. Was the research question stated? | 4 | 4 | 1 | 1 | 1 |
| 2. Was the economic importance of the research question stated? | 4 | 4 | 1 | 1 | 4 |
| 3. Was/were the viewpoint(s) of the analysis clearly stated and justified? | 1 | 1 | 1 | 1 | 1 |
| 4. Was a rationale reported for the choice of the alternative programmes or interventions compared? | 1 | 1 | 1 | 1 | 1 |
| 5. Were the alternatives being compared clearly described? | 1 | 1 | 1 | 1 | 1 |
| 6. Was the form of economic evaluation stated? | 1 | 1 | 1 | 1 | 4 |
| 7. Was the choice of form of economic evaluation justified in relation to the questions addressed? | 2 | 2 | 2 | 2 | 2 |
| 8. Was/were the source(s) of effectiveness estimates used stated? | 1 | 1 | 1 | 1 | 1 |
| 9. Were details of the design and results of the effectiveness study given (if based on a single study)? | 1 | 1 | 1 | 2 | 1 |
| 10. Were details of the methods of synthesis or meta-analysis of estimates given (if based on an overview of a number of effectiveness studies)? | 2 | 2 | 2 | 2 | 2 |
| 11. Were the primary outcome measure(s) for the economic evaluation clearly stated? | 1 | 1 | 1 | 1 | 1 |
| 12. Were the methods used to value health states and other benefits stated? | 2 | 2 | 2 | 1 | 2 |
| 13. Were the details of the subjects from whom valuations were obtained given? | 2 | 2 | 4 | 2 | 2 |
| 14. Were productivity changes (if included) reported separately? | 2 | 2 | 2 | 4 | 4 |
| 15. Was the relevance of productivity changes to the study question discussed? | 2 | 2 | 2 | 4 | 4 |
| 16. Were quantities of resources reported separately from their unit cost? | 1 | 4 | 4 | 4 | 1 |
| 17. Were the methods for the estimation of quantities and unit costs described? | 1 | 1 | 4 | 1 | 1 |
| 18. Were currency and price data recorded? | 1 | 4 | 4 | 1 | 1 |
| 19. Were details of price adjustments for inflation or currency conversion given? | 2 | 2 | 4 | 1 | 1 |
| 20. Were details of any model used given? | 2 | 2 | 2 | 1 | 2 |
| 21. Was there a justification for the choice of model used and the key parameters on which it was based? | 2 | 2 | 2 | 4 |  |
| 22. Was the time horizon of cost and benefits stated? | 1 | 1 | 4 | 1 | 1 |
| 23. Was the discount rate stated? | 4 | 1 | 4 | 1 | 4 |
| 24. Was the choice of rate justified? | 4 | 4 | 2 | 4 | 4 |
| 25. Was an explanation given if cost or benefits were not discounted? | 4 | 2 | 4 | 2 | 4 |
| 26. Were the details of statistical test(s) and confidence intervals given for stochastic data? | 1 | 1 | 2 | 1 | 1 |
| 27. Was the approach to sensitivity analysis described? | 4 | 1 | 4 | 1 | 2 |
| 28. Was the choice of variables for sensitivity analysis justified? | 2 | 1 | 4 | 4 | 4 |
| 29. Were the ranges over which the parameters were varied stated? | 2 | 1 | 2 | 4 | 4 |
| 30. Were relevant alternatives compared? (That is, were appropriate comparisons made when conducting the incremental analysis?) | 1 | 1 | 1 | 1 | 1 |
| 31. Was an incremental analysis reported? | 1 | 1 | 4 | 1 | 1 |
| 32. Were major outcomes presented in a disaggregated as well as aggregated form? | 1 | 1 | 4 | 4 | 4 |
| 33. Was the answer to the study question given? | 1 | 1 | 1 | 1 | 1 |
| 34. Did conclusions follow from the data reported? | 1 | 1 | 1 | 1 | 1 |
| 35. Were conclusions accompanied by the appropriate caveats? | 1 | 1 | 1 | 1 | 1 |
| 36. Were generalisability issues addressed? | 4 | 1 | 4 | 4 | 4 |
