# Supplementary material for: Improving oral health and related health behaviours (substance use, smoking, diet) in people with severe and multiple disadvantage: A systematic review of effectiveness and cost-effectiveness of interventions
Source: PLoS One. 2024 Apr 18;19(4):e0298885. doi: 10.1371/journal.pone.0298885 (PMC11025870; doi:10.1371/journal.pone.0298885)
Supplement: S5 File — (DOCX) [file pone.0298885.s006.docx]

# **Table C. Detailed study characteristics of included studies**

| **Study** | **Country** | **Design** | **Outcome Category** | **Target Population** | **Participant details** |
| --- | --- | --- | --- | --- | --- |
| Aubry  2019 | Canada | RCT | Substance Use | Homeless | N = 201 randomised Intervention:  66% male 18-30 years: 29(29%); 31-40years: 21(21%); 41-50years: 31(31%); 51+years: 20(20%) Control: 63% male 18-30 years: 22(22%); 31-40years: 27(27%); 41-50years: 37(37%); 51+years: 14(14%) |
| Burnam 1995 | USA | RCT | Substance Use | Homeless + Substance Users | N = 276 84% male; age (mean) = 37 |
| Cherner 2017 | Canada | Quasi-experimental | Substance Use | Homeless + Substance Users | N = 178 randomised Intervention:  44.9% male; age [mean (SD)] = 40.06 (9.62) Control: 58.4% male; age [mean (SD)] = 40.04 (9.96) |
| Ciaranello 2006 | USA | Quasi-experimental | Oral Health | Homeless | N = 609 randomised Intervention:  62% male; age [mean (SD)] = 41.6 (9.6) Control: 78% male; age [mean (SD)] = 41.3 (10.4) |
| Collins  2019 | USA | RCT | Substance Use | Homeless + Substance Users | N = 169 randomised 76% male; age [mean (SD)] = 47.86 (9.56) |
| Collins  2021 | USA | RCT | Substance Use | Homeless + Substance Users | N = 308  Intervention 1:  85% male; age [mean (SD)] = 49.27 (9.11)  Intervention 2:  87% male; age [mean (SD)] = 46.55 (10.46)  Intervention 3:  84% male; age [mean (SD)] = 49.38 (7.35)  Control:  79% male; age [mean (SD)] = 47.51 (9.50) |
| Cox  1998 | USA | RCT | Substance Use | Homeless + Substance Users | N = 298 randomised 81% male; age [mean (SD)] = 42.9 (10.6) |
| Drake  1997 | USA | Quasi-experimental | Substance Use | Homeless + Substance Users | N = 217 randomised Intervention:  36.1% male; age [mean (SD)] = 36.2 (6.9) Control: 27.1% male; age [mean (SD)] = 34.4 (7.2) |
| Ferreiro  2022 | Spain | RCT | Substance Use | Homeless + Substance Users | N = 87  Intervention:  85% male; age [mean (SD)] = 50.25 (10.56)  Control:  83% male; age [mean (SD)] = 50.51 (10.84) |
| French  1999 | USA | Quasi-experimental | Substance Use | Homeless + Substance Users | N = 342 randomised no gender/age data reported |
| Hwang  2011 | Canada | Quasi-experimental | Substance Use | Homeless | N = 112 randomised Intervention:  65% male 17-30 years: 9 (20%); 31-40 years: 15 (33%); 41-50 years: 11 (24%); 51-60 years: 10 (22%); ≥61 years: 1 (2%) Control: 77% male 17-30 years: 13 (20%); 31-40 years: 23 (35%); 41-50 years: 18 (27%); 51-60 years: 11 (17%); ≥61 years: 1 (2%) |
| Kashner  2002 | USA | RCT | Substance Use | Homeless + Substance Users | N = 162 randomised Intervention:  age [mean (SD)] = 42.7 (7.2) Control: age [mean (SD)] = 44.4 (7.6) |
| Kirst  2015 | Canada | RCT | Substance Use | Homeless | N = 575 randomised 68.52% male; age [mean (SD)] = 29.80(11.78) |
| Koffarnus 2011 | USA | RCT | Substance Use | Homeless + Substance Users | N = 124 randomised Intervention 1:  79.1% male; age [mean (SD)] = 42.0 (8.5) Intervention 2: 81% male; age [mean (SD)] = 45.2 (8.0) Control: 81.1% male; age [mean (SD)] = 43.0 (7.6) |
| Lam  1995 | USA | RCT | Substance Use | Homeless + Substance Users | N = 294 randomsied Intervention:  100% male; age [mean (SD)] = 32.6(6.8) Control: 100% male; age [mean (SD)] = 32.5(7.5) |
| Loubiere 2022 | France | RCT | Substance Use | Homeless + Substance Users | N = 703 randomsied Intervention:  80.17% male; age [mean (SD)] =38.12 (9.65) Control: 84.86% male; age [mean (SD)] = 39.41(10.29) |
| Malte  2017 | USA | RCT | Substance Use | Homeless + Substance Users | N = 181 randomised Intervention:  97.8% male; age [mean (SD)] = 50.5(0.5) Control: 97.8% male; age [mean (SD)] = 50.7(9.7) |
| Mares 2011 | USA | RCT | Substance Use | Homeless | N = 385 Intervention:  75% male; age [mean (SD)] = 45.2(9.1) Control: 80% male; age [mean (SD)] = 46.1(9.8) |
| Milby 1996 | USA | RCT | Substance Use | Homeless + Substance Users | N = 131 Intervention:  72.5% male; age [mean (SD)] = 36.0(6.6) Control: 87.1% male; age [mean (SD)] = 35.7(6.2) |
| Milby 2000 | USA | RCT | Substance Use | Homeless + Substance Users | N = 110 76% male; age [mean (SD)] = 38.1(7.4) |
| Milby 2005 | USA | RCT | Substance Use | Homeless + Substance Users | N = 196 Intervention 1:  76% male; age [mean (SD)] = 40.9(7.2) Intervention 2: 75% male; age [mean (SD)] = 38.4(6.7) Control: 76% male; age [mean (SD)] = 38.2(7.4) |
| Morse 2008 | USA | Quasi-experimental | Substance Use | Homeless + Substance Users | N = 270 randomised Intervention:  76% male; age [mean (SD)] = 40.02(9.12) Control: no details provided |
| Nyamathi 2017 | USA | RCT | Substance Use | Homeless + Substance Users + Repeat offenders | N = 130 Intervention:  0% male; age [mean (SD)] = 39.1(11.5) Control: 0% male; age [mean (SD)] = 38.6(11.3) |
| O'Campo 2016 | Canada | RCT | Substance Use | Homeless | N = 197 randomised Intervention:  66% male; age [mean (SD)] = 38.18(11.04) Control: 79% male; age [mean (SD)] = 41.9(11.81) |
| Okuyemi 2013 | USA | RCT | Smoking | Homeless | N = 430 Intervention:  73.1% male; age [mean (SD)] = 44.5(9.7) Control: 76.2% male; age [mean (SD)] = 44.42(10.1) |
| Orwin 1994 | USA | Quasi-experimental | Substance Use | Homeless + Substance Users | N not reported Almost half of the clients (46 percent) were between the ages of 25 and 34, and more than four-fifths (82 percent) were between age 25 and 54. Almost one-third (29 percent) were women. |
| Rash 2018 | USA | RCT | Smoking | Homeless | N = 70 randomised Intervention:  73% male; age [mean (SD)] = 44.7 (12.3) Control: 75.8% male; age [mean (SD)] = 45.4 (10.5) |
| Slesnick 2023 | USA | RCT | Substance Use | Homeless + Substance Users | N = 240 randomised 100% female; age [mean (SD)] = 21.57(1.80) |
| Somers 2015 | Canada | RCT | Substance Use | Homeless | **Trial 1** N = 297 randomised Intervention 1:  77% male; age [mean(SD)] = 40.0(11.6) Intervention 2:  74% male; age [mean(SD)] = 39.0(10.8) Control: 71% male; age [mean(SD)] = 39.5(11.2) **Trial 2** N = 200 randomised Intervention:  71% male; age [mean(SD)] = 42.1(10.4) Control: 71% male; age [mean(SD)] = 43.1(10.6) |
| Sosin  1995 | USA | Quasi-experimental | Substance Use | Homeless + Substance Users | N = 419 randomised Intervention 1:  71.5% male; age (mean) = 35.5 Intervention 2: 75.8% male; age (mean) = 35.2 Control: 75.9% male; age (mean) = 34.6 |
| Stahler 1995 | USA | Quasi-experimental | Substance Use | Homeless + Substance Users | N = 722 randomised 100% male Intervention 1:  age (mean) = 32.1 Intervention 2:  age (mean) = 32.8 Control: age (mean) = 32.8 |
| Stockwell 2021 | Canada | Quasi- experimental | Substance Use | Homeless + Substance Users | N = 175 randomised Intervention:  79.6% male; age [mean] = 47.69 Control: 80.17% male; age [mean] = 46.23 |
| Tsai 2010 | USA | Quasi-experimental | Substance Use | Homeless | N = 709 randomised Intervention:  76.5% male; age [mean (SD)] = 46.0(9.2) Control: 73.6% male; age [mean(SD)] = 44.8(10.1) |
| Tsemberis 2004 | USA | RCT | Substance Use | Homeless | N = 225 randomised 79% male 18–30: 39 (19%); 31–40: 59 (29%); 41–50: 62 (30%); 51–60: 36 (17%); ≥61: 10 (5%) average age was 41.3 years |
