# Supplementary material for: Improving oral health and related health behaviours (substance use, smoking, diet) in people with severe and multiple disadvantage: A systematic review of effectiveness and cost-effectiveness of interventions
Source: PLoS One. 2024 Apr 18;19(4):e0298885. doi: 10.1371/journal.pone.0298885 (PMC11025870; doi:10.1371/journal.pone.0298885)
Supplement: S6 File — (DOCX) [file pone.0298885.s007.docx]

# **Fig A. Risk of bias across each domain for each included study**

|  | Random sequence generation | Allocation concealment | Blinding of participants and personnel | Blinding of outcome assessment | Incomplete outcome data | Selective outcome reporting | Other sources of bias |
| --- | --- | --- | --- | --- | --- | --- | --- |
| Aubry 2019 |  |  |  |  |  |  |  |
| Burnam 1995 |  |  |  |  |  |  |  |
| Collins 2019 |  |  |  |  |  |  |  |
| Collins 2021 |  |  |  |  |  |  |  |
| Cox 1998 |  |  |  |  |  |  |  |
| Ferreiro 2022 |  |  |  |  |  |  |  |
| Kashner 2002 |  |  |  |  |  |  |  |
| Kirst 2015 |  |  |  |  |  |  |  |
| Koffarnus 2011 |  |  |  |  |  |  |  |
| Lam 1995 |  |  |  |  |  |  |  |
| Loubiere 2022 |  |  |  |  |  |  |  |
| Malte 2017 |  |  |  |  |  |  |  |
| Milby 1996 |  |  |  |  |  |  |  |
| Milby 2000 |  |  |  |  |  |  |  |
| Milby 2005 |  |  |  |  |  |  |  |
| Nyamathi 2017 |  |  |  |  |  |  |  |
| O'Campo 2016 |  |  |  |  |  |  |  |
| Okuyemi 2013 |  |  |  |  |  |  |  |
| Rash 2017 |  |  |  |  |  |  |  |
| Rash 2018 |  |  |  |  |  |  |  |
| Slesnick 2023 |  |  |  |  |  |  |  |
| Somers 2015 |  |  |  |  |  |  |  |
| Tsemberis 2004 |  |  |  |  |  |  |  |
| Low risk of bias  Unclear  High risk of bias |  |  |  |  |  |  |  |
