# Supplementary material for: Improving oral health and related health behaviours (substance use, smoking, diet) in people with severe and multiple disadvantage: A systematic review of effectiveness and cost-effectiveness of interventions
Source: PLoS One. 2024 Apr 18;19(4):e0298885. doi: 10.1371/journal.pone.0298885 (PMC11025870; doi:10.1371/journal.pone.0298885)
Supplement: S7 File — (DOCX) [file pone.0298885.s008.docx]

| **Table D. CASP assessments for each included study** | **Cherner 2017** | **Ciaranello 2006** | **Drake 1997** | **French 1999** | **Hwang 2011** | **Morse 2008** | **Orwin 1994** | **Sosin 1995** | **Stahler 1995** | **Tsai 2010** | **Stockwell 2021** |
| --- | --- | --- | --- | --- | --- | --- | --- | --- | --- | --- | --- |
| Did the study address a clearly focused issue? | yes | yes | yes | yes | yes | yes | yes | yes | yes | yes | yes |
| Was the cohort recruited in an acceptable way? | yes | yes | yes | yes | yes | yes | yes | yes | yes | yes | yes |
| Was the exposure accurately measured to minimise bias? | yes | yes | yes | yes | yes | yes | yes | yes | yes | yes | yes |
| Was the outcome accurately measured to minimise bias? | no | no | no | no | no | no | no | no | no | no | unclear |
| Have the authors identified all important confounding factors? | no | no | no | yes | yes | no | yes | yes | no | yes | yes |
| Have they taken account of the confounding factors in the design and/or analysis? | no | yes | no | yes | yes | no | yes | yes | no | yes | yes |
| Was the follow up of subjects complete enough? | no | yes | no | unclear | yes | unclear | yes | unclear | yes | yes | unclear |
| Was the follow up of subjects long enough? | yes | yes | yes | yes | yes | yes | unclear | yes | unclear | yes | yes |
| What are the results of this study? |  |  |  |  |  |  |  |  |  |  |  |
| How precise are the results? |  |  |  |  |  |  |  |  |  |  |  |
| Do you believe the results? | yes | yes | yes | yes | yes | yes | yes | yes | yes | yes | yes |
| Can the results be applied to the local population? |  |  |  |  |  |  |  |  |  |  |  |
| Do the results of this study fit with other available evidence? |  |  |  |  |  |  |  |  |  |  |  |
| What are the implications of this study for practice? |  |  |  |  |  |  |  |  |  |  |  |
