# Supplementary material for: Improving oral health and related health behaviours (substance use, smoking, diet) in people with severe and multiple disadvantage: A systematic review of effectiveness and cost-effectiveness of interventions
Source: PLoS One. 2024 Apr 18;19(4):e0298885. doi: 10.1371/journal.pone.0298885 (PMC11025870; doi:10.1371/journal.pone.0298885)
Supplement: S8 File — (DOCX) [file pone.0298885.s009.docx]

# **Table E. Detailed intervention breakdown by study and outcome type**

| **Study** | **Outcome** | **Intervention Grouping** | **Intervention components** | **Intervention description** |
| --- | --- | --- | --- | --- |
| Aubry 2019 | combined | Structural + Community + Individual | Housing, mental health, therapeutic community/ACT | Housing First (HF) with assertive community treatment (ACT) |
| Baggett 2018 | drug + alcohol | Individual | Mental health, financial | Financial incentives (smoking abstinence) nicotine patches and counselling |
| Burnam 1995 | drug + alcohol | Structural + Individual | Housing, mental health, drug/alcohol treatment | Social model residential treatment program - integrated mental health and substance abuse treatment |
|  |  | Individual + Community | Mental health, drug/alcohol treatment | Social model non-residential program - community-based non-residential program using same approach |
| Cherner 2017 | drug + alcohol | Structural + Individual | Housing, mental health, drug/alcohol treatment | Housing First - scattered-site housing and intensive case management services |
| Collins 2019 | alcohol | Individual | Drug/alcohol treatment | Harm Reduction Treatment for Alcohol - a) collaborative tracking of participant-preferred alcohol-related metrics, b) elicitation of participants’ own harm reduction and/or QoL goals as the primary treatment focus, and c) discussion of safer drinking strategies. |
| Cox 1998 | alcohol | Structural + Individual | Housing, drug/alcohol treatment, financial | Intensive case management - housing, financial, substance use reduction |
| Drake 1997 | drug + alcohol + combined | Structural + Individual | Housing, mental health, drug/alcohol treatment | Integrated treatment - mental health, substance abuse, and housing interventions |
| French 1999 | drug + alcohol | Structural + Individual + Community | Housing, mental health, drug/alcohol treatment | Modified therapeutic community (TC) intervention |
| Hwang 2011 | drug + alcohol | Structural + Individual | Housing, mental health | supported housing intervention - the program partnered with a mental health and community support services organization that provided onsite support to tenants. Specifically, tenant support workers assisted residents with mental illness to transition into the housing program as well as providing ongoing help with living skills, counselling, and advocacy) |
| Kashner 2002 | drug + alcohol | Structural + Individual | Work, drug/alcohol treatment | Department of veterans affairs compensated work therapy program (CWT), a clinician-supervised, abstinence-contingent, performance-based work program - offers pay rewards requires adherence to a schedule of outpatient addiction services. Control group had access to comprehensive health services including addictions treatment and vocational rehabilitation |
| Kirst 2015 | drug + alcohol + combined | Structural + Individual | Housing, mental health | Housing First vs TAU |
| Koffarnus 2011 | alcohol | Structural + Individual | Work, drug/alcohol treatment | Therapeutic Workplace (substance abuse intervention that promotes abstinence while simultaneously addressing the issues of poverty and lack of job skills). Conditions: requiring abstinence from alcohol to engage in paid job skills training (Contingent Paid Training group), offering paid job skills training with no abstinence contingencies (Paid Training group) or offering unpaid job skill training with no abstinence contingencies (Unpaid Training group). |
| Lam 1995 | drug + alcohol | Structural + Individual | Housing, work, mental health, drug/alcohol treatment | Shelter-based treatment program…consisting of 90 days residential treatment and 6 months aftercare (intervention - case management (focus on housing/employment), group therapy, individual counselling, NA/AA) vs 'usual services' (homeless shelters w/ case workers who sought to counsel the men into appropriate services and programs, including drug treatment and vocation training programs) |
| Loubiere 2022 | alcohol + combined | Structural + Individual + Community | Housing, mental health, drug/alcohol treatment | French Housing First (HF) - Independent housing with Assertive community therapy (ACT) vs TAU |
| Malte 2017 | drug + alcohol + combined | Structural + Individual | Housing, drug/alcohol treatment | intensive addiction/housing case management (AHCM) vs housing support group (HSG) control |
| Mares 2011 | drug + alcohol | Structural + Individual + Community | Housing, healthcare | Comprehensive Housing and Health Care Services Versus Usual Local Care |
| Milby 1996 | drug + alcohol | Structural + Individual | Housing, mental health, drug/alcohol treatment | enhanced day treatment program plus abstinent contingent work therapy and housing (EC) vs usual care |
| Milby 2000 | combined | Structural + Individual | Housing, mental health, drug/alcohol treatment, work | behavioral day treatment plus abstinence contingent housing and work therapy (DT + ) versus behavioral day treatment alone (DT). |
| Milby 2005 | combined | Structural + Individual | Housing, mental health | day treatment and no housing (NH), housing contingent on drug abstinence (ACH), or housing not contingent on abstinence (NACH). |
| Morse 2008 | drug + alcohol | Individual + Community | Mental health | standard care (SC), assertive community treatment only (ACTO), integrated assertive community treatment (IACT), and new integrated assertive community treatment (NIACT). |
| Nyamathi 2017 | drug + alcohol + combined | Individual | Mental health | dialectical behavioral therapy-corrections modified (DBT-CM) program vs health promotion (HP) program |
| O'Campo 2016 | combined | Structural + Individual + Community | Housing, mental health | Housing First and assertive community treatment team (HF+ACT) or treatment as usual (TAU) |
| Orwin 1994 | alcohol | Structural + Individual | Housing, drug/alcohol treatment | NIAAA COMMUNITY DEMONSTRATION PROGRAM - In addition toproviding treatment, the projects developed innovative approaches to servingthe target population. These ranged from outreach programs in the streets andhomeless shelters to intensive case management and supportive housing arrangements. Thus, in contrast to more traditional demonstration projects, theCommunity Demonstration Program was designed to explore the usefulness ofmany diverse treatment models rather than to demonstrate a single treatment model in different settings |
| Slesnick 2023 | combined | Structural + Individual | Housing, mental health, drug/alcohol treatment, healthcare | Integrated housing services with mental health and substance use therapy vs. services as usual |
| Somers 2015 | combined | Structural + Individual | Housing, mental health, drug/alcohol treatment | HF and TAU |
| Sosin 1995 | combined | Structural + Individual | Housing, mental health, drug/alcohol treatment | Arm 1: case management, Arm 2: case management + supported housing |
| Stahler 1995 | drug + alcohol | Structural + Individual | Housing, healthcare, mental health | Group 1 - integrated comprehensive residential services; Group 2 - on-site shelter-based intensive case management with referrals to community services; Group 3 - usual care shelter services with case management |
| Stockwell 2021 | alcohol | Structural + Individual + Community | Housing, mental health, drug/alcohol treatment, healthcare | Managed Alcohol Programs vs. Usual care |
| Tsai 2010 | drug + alcohol | Structural + Individual | Housing, mental health | “Housing first” versus “residential treatment first” - residential treatment or transitional housing before being placed into independent housing (RTF vs IHF) residential treatment first vs independent housing first |
| Tsemberis 2004 | drug + alcohol | Structural + Individual + Community | Mental health, drug/alcohol treatment | housing contingent on treatment and sobriety (control) vs immediate housing without treatment prerequisites (experimental) (HF) |
